# How robust are findings of pairwise and network meta-analysis in the presence of missing participant outcome data?

**DOI:** 10.1186/s12916-021-02195-y

**Published:** 2021-12-21

**Authors:** Loukia M. Spineli, Chrysostomos Kalyvas, Katerina Papadimitropoulou

**Affiliations:** 1 Midwifery Research and Education Unit, Hannover Medical School, Hannover, Germany; 2 Biostatistics and Research Decision Sciences, MSD Europe Inc., Brussels, Belgium; 3 Clinical Epidemiology, Leiden University Medical Center, Leiden, The Netherlands; 4 Data Science and Biometrics, Danone Nutricia Research, Utrecht, The Netherlands

**Keywords:** Missing outcome data, Systematic reviews, Sensitivity analysis, Robust conclusions, Pattern-mixture model, Bayesian analysis

## Abstract

**Background:**

To investigate the prevalence of robust conclusions in systematic reviews addressing missing (participant) outcome data via a novel framework of sensitivity analyses and examine the agreement with the current sensitivity analysis standards.

**Methods:**

We performed an empirical study on systematic reviews with two or more interventions. Pairwise meta-analyses (PMA) and network meta-analyses (NMA) were identified from empirical studies on the reporting and handling of missing outcome data in systematic reviews. PMAs with at least three studies and NMAs with at least three interventions on one primary outcome were considered eligible. We applied Bayesian methods to obtain the summary effect estimates whilst modelling missing outcome data under the missing-at-random assumption and different assumptions about the missingness mechanism in the compared interventions. The odds ratio in the logarithmic scale was considered for the binary outcomes and the standardised mean difference for the continuous outcomes. We calculated the proportion of primary analyses with robust and frail conclusions, quantified by our proposed metric, the robustness index (RI), and current sensitivity analysis standards. Cohen’s kappa statistic was used to measure the agreement between the conclusions derived by the RI and the current sensitivity analysis standards.

**Results:**

One hundred eight PMAs and 34 NMAs were considered. When studies with a substantial number of missing outcome data dominated the analyses, the number of frail conclusions increased. The RI indicated that 59% of the analyses failed to demonstrate robustness compared to 39% when the current sensitivity analysis standards were employed. Comparing the RI with the current sensitivity analysis standards revealed that two in five analyses yielded contradictory conclusions concerning the robustness of the primary analysis results.

**Conclusions:**

Compared with the current sensitivity analysis standards, the RI offers an explicit definition of similar results and does not unduly rely on statistical significance. Hence, it may safeguard against possible spurious conclusions regarding the robustness of the primary analysis results.

**Supplementary Information:**

The online version contains supplementary material available at 10.1186/s12916-021-02195-y.

## Background

The ubiquity of participant losses (also known as missing participant outcome data, MOD) in systematic reviews in healthcare is well-acknowledged in the literature [1–3]. The inclusion of studies with MOD in systematic reviews further complicates their quantitative synthesis [4, 5]. As the term indicates, MOD refer to unavailable information about the outcome of participants due to several reasons [6]. Addressing MOD, therefore, rests entirely on untestable assumptions on the possible outcome of the missing participants [4, 7]. Kahale et al. [8] reported that imposing clinically implausible assumptions on the outcome of missing participants led to great variation in the summary effect estimates and contradictory conclusions, whilst clinically plausible assumptions mitigated the variability in the summary effect estimates.

Handling MOD in systematic reviews requires an attentive plan to ensure credible results. The Cochrane Handbook promotes sensitivity analyses as necessary means to safeguard against spurious inferences [9]. The authors of systematic reviews are advised to explore how sensitive the results are to different yet reasonable assumptions about MOD in the compared interventions [9]. However, recent evidence on the planning and conduct of sensitivity analysis related to MOD in systematic reviews is underwhelming. Spineli et al. [1] reported that two in five reviews that made their protocol available provided a plan to address MOD in the analysis. Eventually, only 6% of the reviews with MOD in the included studies performed a sensitivity analysis [1]. According to Kahale et al. [2], only 9% of the reviews reported having performed sensitivity analyses related to MOD, with approximately half of them reporting the actual sensitivity analysis results.

We recently proposed a novel framework in the context of sensitivity analyses to objectively infer the robustness of the primary analysis results to different plausible assumptions about the MOD mechanisms [10]. This framework introduces the robustness index (RI) to quantify the similarity of the summary effect estimates from a series of sensitivity analyses to the primary analysis. When the RI does not exceed a pre-specified threshold (a minimally allowed deviation between the primary analysis results and alternative re-analyses), we can deem the primary analysis results robust to a possible risk of bias associated with MOD. Contrary to current sensitivity analysis standards, the RI incorporates a formal definition of ‘similar’ results and does not unduly rely on the statistical significance of the summary effect estimates.

We aim to demonstrate the ease of applying the RI using a collection of published systematic reviews with two or more interventions across several healthcare fields. By calculating the RI, we uncover the prevalence of primary analyses with frail conclusions that translates to a high risk of biased results due to MOD. We also investigate the agreement between RI and the current sensitivity analysis standards, which rely on statistical significance. With this empirical study, we aspire to initiate a paradigm shift in the analysis of aggregate MOD where sensitivity analysis and objective judgement of robustness become state of the art in systematic reviews.

## Methods

### Design

The present empirical study is based on 108 pairwise meta-analyses (PMAs) comprised of at least three studies and 34 network meta-analyses (NMAs) of at least three interventions. The PMAs are part of a broader collection of 140 Cochrane systematic reviews from three review groups on the mental health field published between 01/2009 and 12/2012, assessing both binary and continuous outcomes [3]. The PMAs reported the number of MOD in both arms of every study: 95 (88%) assessed a binary primary outcome, and 13 (12%) a continuous primary outcome. The NMAs are part of a broader collection of 387 systematic reviews of three or more interventions from several healthcare fields published between 01/2009 and 03/2017 [1]. Twenty-nine NMAs (85%) assessed a binary primary outcome, and five (15%) assessed a continuous primary outcome. All NMAs reported the number of MOD in all arms of every study. Additional files 1 and 2 list the systematic reviews with the PMAs and NMAs, respectively, considered in the present work.

### Data extraction

For the binary outcomes, we extracted the number of observed events, MOD, and randomised participants in each study-arm. For the continuous outcomes, we extracted the observed mean outcome and standard deviation, the number of MOD, and randomised participants in each study-arm. The accuracy of the extracted data was heavily dependent on the reporting quality of the eligible systematic reviews, as we did not retrieve the original reports of the corresponding studies.

### Data analysis

#### The pattern-mixture model to handle MOD

To investigate and quantify the robustness of primary analysis results, we conducted various sensitivity analyses by modelling MOD via the pattern-mixture model. This sophisticated model offers the advantage of maintaining the randomised sample of the studies in the analysis, therefore, conforming with the intention-to-treat principle, which is generally preferred in the synthesis of studies [7]. Suppose we have retrieved the reports of *N* studies comparing different sets of interventions {*A*, *B*, *C*, …} for the same target population and condition. We have collected information on the observed aggregate outcome of participants who completed the study (called completers), and the number of MOD, *m*_*ik*_, out of the number randomised, *n*_*ik*_, in arm *k* of study *i*. For a binary outcome, the number of events given the *n*_*ik*_ − *m*_*ik*_ completers and the number of MOD in arm *k* of study *i* are sampled from the corresponding binomial distributions [11]:

${r}_{ik}\sim Bin\left({\theta}_{ik}^o,{n}_{ik}-{m}_{ik}\right)$ and *m*_*ik*_~*Bin*(*q*_*ik*_, *n*_*ik*_)

where ${\theta}_{ik}^o$ and *q*_*ik*_ are the underlying probability of observing an event given the completers and the probability of MOD, respectively. In the case of a continuous outcome, the observed mean outcome in arm *k* of study *i* follows a normal distribution:

$$ {y}_{ik}\sim N\left({\theta}_{ik}^o,{v}_{ik}\right) $$

where ${\theta}_{ik}^o$ and *v*_*ik*_ are the underlying mean outcome given the completers and the variance of the observed outcome (typically assumed known), respectively.

Then, the underlying outcome (i.e. the probability of an event or the mean outcome given the randomised participants) is specified via the pattern-mixture model as follows:

$$ {\theta}_{ik}={\theta}_{ik}^o\bullet \left(1-{q}_{ik}\right)+{\theta}_{ik}^m\bullet {q}_{ik} $$

where ${\theta}_{ik}^m$ refers to the underlying unobserved outcome in the missing participants. For the unobserved outcome, clinically plausible assumptions regarding its relationship to the outcome in the observed participants are made. This relationship is measured using the informative missingness odds ratio (IMOR) parameter for binary outcomes [4, 5] and the informative missingness difference of means (IMDoM) parameter for continuous outcomes [12].

#### Informative missingness odds ratio (IMOR)

The IMOR in arm *k* of study *i* is defined as a function of ${\theta}_{ik}^m$ and ${\theta}_{ik}^o$ as follows:

$$ {e}^{\varphi_{ik}}=\frac{\theta_{ik}^m/\left(1-{\theta}_{ik}^m\right)}{\theta_{ik}^0/\left(1-{\theta}_{ik}^0\right)} $$

The IMOR takes positive values, similar to the odds ratio (OR). IMOR equal to one (*φ*_*ik*_ = 0) translates to the missing-at-random (MAR) assumption and a value different from one (*φ*_*ik*_ ≠ 0) to informative missingness; that is, the unobserved outcomes may be related to their underlying values. For example, for the binary outcome ‘symptom improvement’, IMOR > 1 (*φ*_*ik*_ > 0) indicates that participants who left the study prematurely are more likely to have experienced improvement in their symptoms than participants who completed that intervention.

#### Informative missingness difference of means (IMDoM)

The IMDoM in arm *k* of study *i* is also defined as a function of ${\theta}_{ik}^m$ and ${\theta}_{ik}^o$ using the following formula:

$$ {\psi}_{ik}={\theta}_{ik}^m-{\theta}_{ik}^o $$

The IMDoM takes values from minus to plus infinity; a value different from zero implies informative missingness, and a value equal to zero corresponds to the MAR assumption. A positive IMDoM indicates that a larger outcome on average is more likely to occur in missing participants than in completers, and a negative IMDoM indicates the opposite.

The values of *φ*_*ik*_ and *ψ*_*ik*_ are naturally unknown; thus, one needs to suggest plausible values for these parameters. By convention, we assigned a normal distribution on *φ*_*ik*_ and *ψ*_*ik*_,

$$ {\varphi}_{ik},{\psi}_{ik}\sim N\left({\lambda}_{ik},{\sigma}_{ik}^2\right) $$

where *λ*_*ik*_ reflects our prior belief about the missingness mechanism, and ${\sigma}_{ik}^2$ indicates the uncertainty about our belief [11, 12]. Following the relevant literature, we considered ${\sigma}_{ik}^2={\sigma}^2$ equal to 1 for *φ*_*ik*_ and *ψ*_*ik*_ [4, 11, 12]. Assigning a normal distribution is a better approach to fixing either parameter to an assumed value, which effectively corresponds to imputation [5]. Both pattern-mixture model and imputation maintain the randomised sample. However, by assigning a probability distribution on the unknown parameters, we fully acknowledge the uncertainty about the parameters’ true value. This approach is natural under the Bayesian framework. In contrast, imputation discounts the uncertainty of the assumed value, therefore leading to spuriously precise results [5]. In the present work, we specified *λ*_*ik*_ to be different for the experimental and control arms of a study but same across the corresponding studies, which corresponds to assuming that different interventions may trigger a different missingness mechanism on average. This corresponds to ${\lambda}_{ik}={\lambda}_{t_{ik}}$ where *t*_*ik*_ refers to the intervention investigated in the arm *k* of study *i* (*t*_*ik*_ ∈ {*A*, *B*, *C*, …}). We provide detailed information on the specification of the Bayesian models (e.g. prior distributions and diagnostic evaluation of convergence) in Additional file 3: Note S1 [11–21]. In the following section, we present the values for ${\lambda}_{t_{ik}}$, separately, for log IMOR and IMDoM, which we adopted for the sensitivity analysis.

#### Selection of assumptions for the MOD mechanisms in each intervention

We considered the MAR assumption for the primary analysis (i.e. ${\lambda}_{t_{ik}}=0$) as a plausible reference point when the reasons for MOD are not available for every study [5]. For sensitivity analysis, we defined a set of stringent yet clinically plausible assumptions for ${\lambda}_{t_{ik}}$ without consulting clinical expertise. Our decision was merely logistical; our dataset includes various outcomes and interventions from different healthcare fields. Thus, we would need to involve a great number of experts from each field. Our proposed values, however, are in line with relevant work for aggregate MOD [10, 12]. Specifically, for the IMOR parameter, we allowed $\exp \left({\lambda}_{t_{ik}}\right)$ to take the values 1/3, 1/2, 2, and 3. For the IMDoM parameter, we allowed ${\lambda}_{t_{ik}}$to take the values −2, −1, 1, and 2. For example, in the IMOR scale, $\exp \left({\lambda}_{t_{ik}}\right)$ equal to 1/3 indicates that the odds of an event are three times more likely in completers than in missing participants who received the intervention *t*_*ik*_, whilst $\exp \left({\lambda}_{t_{ik}}\right)$ equal to 3 indicates the opposite for the same intervention. Similarly, in the IMDoM scale, ${\lambda}_{t_{ik}}$equal to −2 indicates that the outcome increases by two units on average in completers than in missing participants who received the intervention *t*_*ik*_, whilst ${\lambda}_{t_{ik}}$equal to 2 indicates the opposite for the same intervention.

Recall that the values for ${\lambda}_{t_{ik}}$mentioned above refer to the intervention investigated in the arm *k* of study *i*. It is possible to assign identical or different ${\lambda}_{t_{ik}}$values to the interventions compared in the same study. For a pairwise comparison, the possible combinations of these values and the value for the MAR assumption yield 5 × 5 assumptions. Table 1 illustrates the 25 assumptions (one for the primary analysis and 24 for sensitivity analyses) in the active and control arms of a two-arm study. We used the same assumptions for all studies in a PMA.

**Table 1:** Assumptions for the missingness mechanisms in a two-arm study

Assumption	IMDoM values		IMOR values	
	Active arm	Control arm	Active arm	Control arm
1	− 2	− 2	1/3	1/3
2	− 2	− 1	1/3	1/2
…	…	…	…	…
5	− 2	2	1/3	3
…	…	…	…	…
13 (MAR)	0	0	1	1
…	…	…	…	…
21	2	− 2	3	1/3
…	…	…	…	…
24	2	1	3	2
25	2	2	3	3

*IMDoM* informative missingness difference of means, *IMOR* informative missingness odds ratio

The same concept applies to an NMA for star-shaped networks because the common anchor intervention is the ‘control arm’ in all studies. These pairwise assumptions are not immediately applicable to a non-star-shaped network where an intervention may be the ‘active arm’ in a study but the ‘control arm’ in another study. In this case, for each assumption, we assigned the ‘control arm’ values to the selected reference intervention and the ‘active arm’ values to the remaining interventions in that network [10]. Therefore, in a non-star-shaped network, the non-reference interventions receive the same assumptions. The reference intervention receives either the same or different assumptions with the non-reference interventions (Table 2).

**Table 2:** Assumptions for the missingness mechanisms in a fictional triangle network

Assumption	IMDoM values			IMOR values		
	A	B	C^a^	A	B	C^a^
1	− 2	− 2	− 2	1/3	1/3	1/3
2	− 2	− 2	− 1	1/3	1/3	1/2
…	…	…	…	…	…	…
13 (MAR)	0	0	0	1	1	1
…	…	…	…	…	…	…
24	2	2	1	3	3	2
25	2	2	2	3	3	3

*IMDoM*, informative missingness difference of means; *IMOR*, informative missingness odds ratio

^a^The reference intervention of the network

#### The robustness index (RI)

To quantify the (dis)similarity between the primary analysis results (under MAR assumption) and the results from the 24 sensitivity analyses, we calculated the RI, a metric we recently proposed [10], which considers the magnitude and standard error of the summary effect estimate(s) in primary and sensitivity analyses as follows:

$$ \mathrm{RI}=\sqrt{\sum \limits_{i\in A}{D}_i^2}=\sqrt{D_1^2+{D}_2^2+\dots +{D}_{12}^2+{D}_{14}^2\dots +{D}_{25}^2} $$

where *A* = {1, 2, …12, 14, …, 25} refers to the |*A*| = 24 informative assumptions about the ${\lambda}_{t_{ik}}$ in Table 1 (one assumption per sensitivity analysis), and *D*_*i*_ is the Kullback-Leibler divergence measure [22] for two normal distributions,

$$ {D}_i=\frac{1}{2}\left[{\left(\frac{s_{13}}{s_i}\right)}^2+\frac{{\left({\hat{\mu}}_i-{\hat{\mu}}_{13}\right)}^2}{s_i^2}-1+2\times \mathit{\ln}\left(\frac{s_i}{s_{13}}\right)\right] $$

with ${\hat{\mu}}_i$ and ${\hat{\mu}}_{13}$ being the summary effect estimates under the assumption *i* and primary analysis (the MAR assumption has the number 13 in Table 1), respectively, and *s*_*i*_ and *s*_13_ being the corresponding standard errors. In the Bayesian analysis, ${\hat{\mu}}_i$ and *s*_*i*_ refer to the posterior mean and the posterior standard deviation of the summary effect estimate, respectively. The RI ranges from zero to infinity; a zero value implies a perfect overlap between the distributions of summary effects under MAR and alternative re-analyses. The lower the value of the RI, the larger the proximity between the distributions, and thus, approximating the MAR assumption with any informative assumption would not materially change the conclusions [10]. Note that the RI is comparison-specific; we can calculate as many RIs as the number of pairwise comparisons. It follows then that for a PMA, one RI is calculated, and for an NMA, the number of RIs equals *T*(*T* − 1)/2, where *T* is the number of interventions in the network.

#### The threshold of robustness

The interpretation of the RI requires a threshold to which the values of the index are contrasted. As a threshold of robustness, we used one standard deviation of low statistical heterogeneity [10]. Low statistical heterogeneity was defined as the median of the empirically based distribution for the between-study variance (*τ*^2^) in the case of a general healthcare setting [16, 17]. For a binary outcome, this median equals 0.08 in the log OR scale, and for a continuous outcome, 0.03 in the standardised mean difference (SMD) scale. Therefore, for a given comparison, an RI value less than $\sqrt{0.08}=0.28$ (1.32 after exponentiation) or $\sqrt{0.03}=0.17$ infers robustness, and a value at least 0.28 or 0.17 implies a lack of robustness in the log OR and SMD scale, respectively [10]. We used these thresholds for all PMAs/NMAs of our dataset. To infer the robustness of the whole network, we considered the following decision framework: when the RI equals or exceeds the robustness threshold for at least one comparison, we conclude a lack of robustness in the network [10]. Robustness can be claimed for a network when the RI is less than the robustness threshold for all possible comparisons [10].

#### Application to a network with a considerable risk of bias due to MOD

**Fig. 1:**
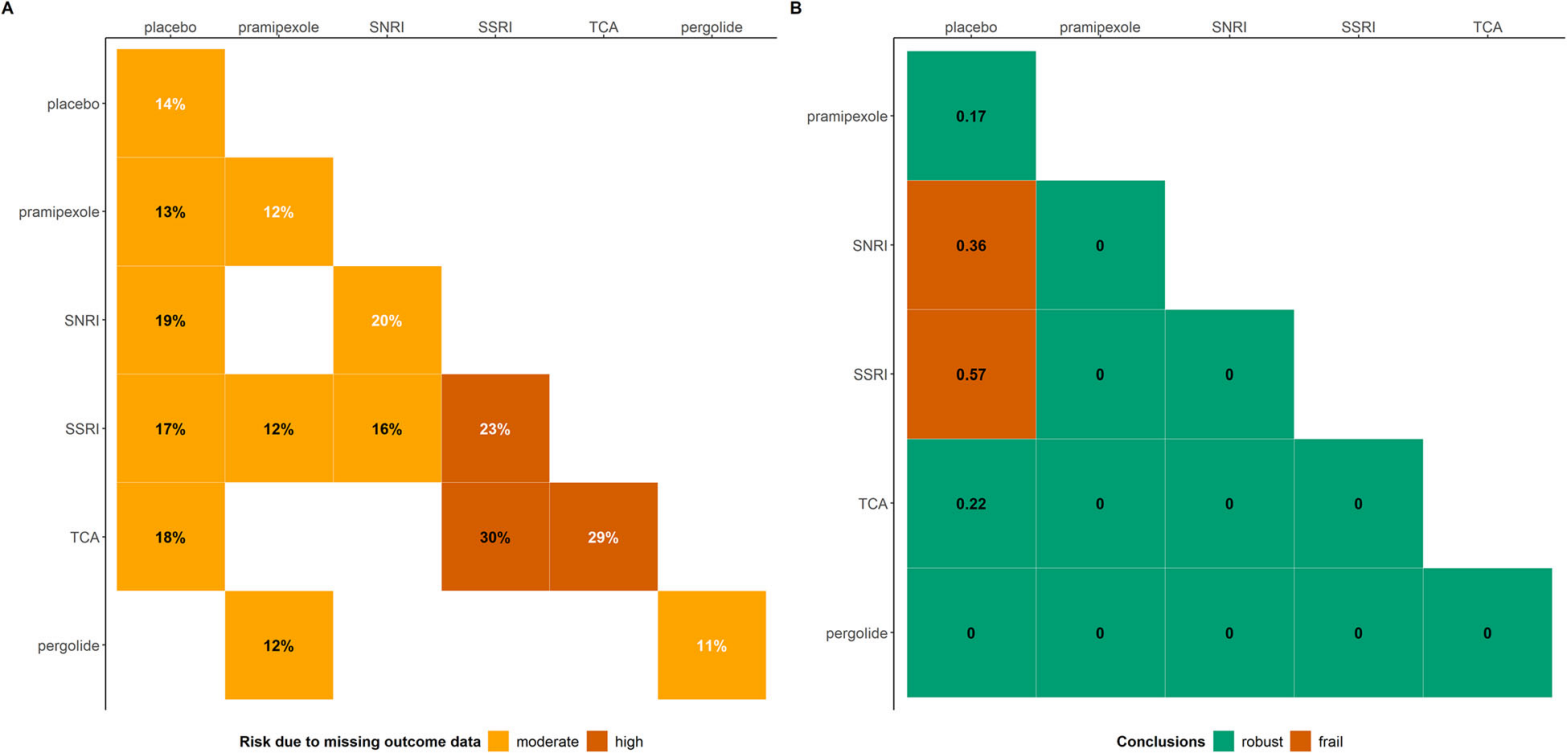
**A** Heatmap of the proportion of total MOD in each intervention (percentages in white, main diagonal) and observed comparison (percentages in black, lower off-diagonal) in the network of antidepressants for participants with Parkinson’s disease [23]. **B** Heatmap of the robustness index (RI) values for every possible comparison in the network of antidepressants for participants with Parkinson’s disease [23]. The pairwise comparisons are read from left to right. Red cells indicate a lack of robustness (RI ≥ 0.28), and green cells indicate the robustness (RI < 0.28) of the primary analysis results for the corresponding comparison

We illustrate the sensitivity analysis framework and calculation of the RI in a network of multiple interventions using a systematic review of five antidepressants and a placebo to relieve the symptoms of depression in participants with Parkinson's disease (Additional file 4: Figure S1) [23]. The authors defined relief of symptoms as a reduction of at least 50% from the baseline score on various scales for depression assessment (binary outcome) [23]. Figure 1A shows the percentage of total MOD in each intervention (percentages in white, main diagonal) and pairwise comparison (percentages in black, lower off-diagonal), respectively. The percentage of total MOD in an intervention is the ratio of the sum of MOD for that intervention across the corresponding studies to the sum of the randomised participants in that intervention. Similarly follows the definition of the percentage of total MOD in an observed comparison. Overall, the percentage of total MOD in all pairwise comparisons and interventions exceeded 5%, the threshold of low risk of bias due to MOD [24]. The comparison of TCA with SSRI and the interventions thereof had at least 20% of total MOD, the threshold of high risk of bias due to MOD [24].

Figure 1B is a heatmap of the RI values for all possible comparisons in the network. Robustness could not be inferred for SNRI and SSRI versus placebo (red cells) since the RI values exceeded the threshold of 0.28. The credibility of the primary analysis results for this network is, thus, overall questionable. Interestingly, the comparisons between antidepressants resulted in zero RI values (after rounding to the second decimal). This may be explained partly due to (a) sharing the same assumptions about the missingness mechanisms (contrary to comparisons with placebo) and (b) the consistency equation that warrants the agreement between indirect and direct evidence for a given comparison, as has been recently shown [25]. These comparisons were obtained as linear combinations of the placebo comparisons via the consistency equation. Therefore, after adjusting for MOD via the pattern-mixture model, any residual bias in the placebo comparisons may have been mitigated in the remaining comparisons.

#### Investigating the risk of frail conclusions in PMAs and NMAs

We investigated the proportion of PMAs and NMAs with questionable conclusions in association with the extent of MOD. A study is associated with a low, moderate, or high risk of bias due to MOD when the corresponding proportion of total MOD is up to 5%, more than 5% and up to 20%, or more than 20%, respectively [24]. Since the number of MOD differs from study to study, it is not straightforward to characterise a pairwise comparison as having a low, moderate, or high risk of bias due to MOD. In an NMA, where we deal with many studies comparing different interventions, labelling a network as having a low, moderate, or high risk of bias due to MOD is unarguably more challenging.

For each PMA and NMA, we counted the number of studies with a low, moderate, and high risk of bias due to MOD and then, we grouped them into one of the following three categories: analyses with (a) more studies with low risk, (b) more studies with moderate risk, and (c) more studies with high risk. Those PMAs/NMAs with an equal number of studies in at least two of the risk levels (i.e. low, moderate, and high) were placed in the second category. Stacked barplots were used to illustrate the percentage of PMAs/NMAs with and without questionable conclusions (based on the RI) within each category. Violin plots and boxplots were also created to describe the distribution of the RI in each group in PMAs and NMAs, respectively. In the NMAs, we considered the maximum RI among the comparisons of the corresponding analysed network to create the boxplots. The results for PMA and NMA are presented separately. All plots were created using the R-package *ggplot2* [26]. The R-package *gghalves* was used to integrate dots in the violin plots and boxplots [27].

#### Investigating the agreement with the current sensitivity analysis standards

We investigated the proportion of PMAs and NMAs that reached the same conclusions under our sensitivity analysis framework and the current sensitivity analysis standards. Thus, we examined whether the statistical significance of the summary effect estimate of a pairwise comparison under the primary analysis changed in any of the subsequent 24 re-analyses. A 95% credible interval of the summary effect estimate that excludes the threshold for null effect implies statistical significance for that comparison. If the statistical significance under the primary analysis changed in *at least one* re-analysis, we concluded the corresponding comparison to be frail under the current sensitivity analysis standards. For the NMA, we followed the same decision framework as before: if one summary estimate of a pairwise comparison in the network changed statistical significance, the NMA findings were deemed to be frail.

We tabulated the percentage of PMAs/NMAs with the same or different conclusions under these two sensitivity analysis frameworks. We used Cohen’s kappa statistic (*k*) to measure the agreement between the conclusions of our proposed framework and the current sensitivity analysis standards [28]. The thresholds of agreement proposed by Landis and Koch [29] were adopted to interpret the Cohen’s kappa statistic: *k* < 0 indicates poor agreement, 0 < *k* ≤ 0.20 implies slight agreement, 0.20 < *k* ≤ 0.40 implies fair agreement, 0.40 < *k* ≤ 0.60 indicates a moderate agreement, 0.60 < *k* ≤ 0.80 indicates a substantial agreement, and 0.80 < *k* ≤ 1.00 indicates an almost perfect agreement. We reported the estimated statistic and 95% confidence interval (CI) separately for PMAs and NMAs. The R-package *fmsb* [30] was used to obtain Cohen’s kappa statistic and 95% CI and the R-package *caret* [31] to create the confusion matrices. All functions and data related to this manuscript are publicly available at https://github.com/LoukiaSpin/Empirical-Evidence-on-Robustness-in-Meta-analyses.git.

## Results

### Characteristics of the dataset

A total of 108 PMAs (95 on binary and 13 on continuous primary outcomes) and 34 NMAs (29 on binary and five on continuous primary outcomes) comprised the analysed dataset (Table 3). NMAs included inherently more studies than PMAs (median 14 and 4, respectively) with a substantially larger randomised sample (median 247 and 60, respectively). However, the total sample size ranged from very few participants (4 and 12 in PMA and NMA, respectively) to a few thousands (1996 and 18,201 in PMA and NMA, respectively) in both analyses. The number of investigated interventions and the percentage of observed comparisons varied considerably across the analysed networks (range 3 to 22 and 13 to 100%, respectively). Overall, the event frequency observed in study-arms indicated that both analyses dealt mostly with a frequent binary outcome (Table 3). Only 33 PMAs (31%) and nine NMAs (26%) included at least one study with zero events or non-events. Most NMAs investigated comparisons with placebo (68% of NMAs versus 34% of PMAs). PMAs included mostly comparisons among pharmacological interventions (43% of PMAs versus 26% of NMAs). Comparisons with non-pharmacological interventions were the least prevalent intervention-comparison type in the dataset (23% of PMAs and 6% of NMAs). Studies with a high risk of bias due to MOD were predominant in PMAs (median 55%); 8% of the PMAs comprised such studies only. On the contrary, NMAs included mostly studies with a low risk of bias due to MOD (median 50%), followed by studies with a moderate risk of bias due to MOD (median 44%).

**Table 3:** Characteristics of the 108 pairwise meta-analyses and 34 network meta-analyses. Values are median (range) [number of pairwise meta-analyses or network meta-analyses] unless stated otherwise

Characteristic	PMA	NMA
Number of studies	4 (3 to 25)	14 (4 to 104)
Randomised sample	60 (4 to 1996)	247 (12 to 18201)
Number of interventions	2	6 (3 to 22)
Observed comparisons (%)	1	42 (13 to 100^a^)
Εvent frequency (%) in study-arms (binary outcomes only)	47 (26 to 67)^b^	60 (42 to 76)^b^
Studies with at least one zero-cell (binary outcomes only)	2 (1 to 6) [33]	1 (1 to 4)[9]
Intervention-comparison type:		
Pharma versus placebo^c^	37 (34)	23 (68)
Pharma versus pharma^c^	46 (43)	9 (26)
Non-pharma^d^ versus pharma^c^	3 (3)	1 (3)
Non-pharma versus non-pharma^c^	22 (20)	1 (3)
Proportion of studies associated with:		
Low risk of bias due to MOD^e^	33 (8 to 100) [6]^f^	50 (6 to 100) [3]^f^
Moderate risk of bias due to MOD^e^	33 (10 to 100) [2]^f^	44 (7 to 92)
High risk of bias due to MOD^e^	55 (11 to 100) [9]^f^	28 (4 to 80)

*MOD* missing participant outcome data, *NMA* network meta-analysis, *pharma* pharmacological interventions, *PMA* pairwise meta-analysis

^a^Two networks on the continuous outcome are closed triangles

^b^Values are median (interquartile range). The range of event frequency (%) in study-arms was 0 to 100 in pairwise meta-analyses and network meta-analyses

^c^Values are numbers (percentages)

^d^Non-pharmacological interventions include medical devices, surgical, complex, resources and infrastructure, behavioural, psychological, physical, complementary, educational, radiotherapy, vaccines, cellular and gene and screening [32]

^e^Following the classification by Sackett et al. [24]: a proportion of missing participants up to 5% implies a low risk of bias due to MOD, more than 5% and up to 20% indicates a moderate risk of bias due to MOD, and more than 20% indicates a high risk of bias due to MOD

^f^Number of PMAs/NMAs that include only studies with a specific risk of bias due to MOD

### Exclusion due to convergence issues

We excluded one PMA and one NMA on a binary outcome due to convergence problems (Additional file 5: Tables S1 and S2) [33, 34]. Therefore, the final analyses were based on 107 PMAs (94 on binary and 13 on continuous primary outcomes) and 33 NMAs (28 on binary and five on continuous primary outcomes). More details on the reasons for non-convergence can be found in Additional file 3: Note S2 [33–35].

### The risk of frail conclusions in PMA and NMA

**Fig. 2:**
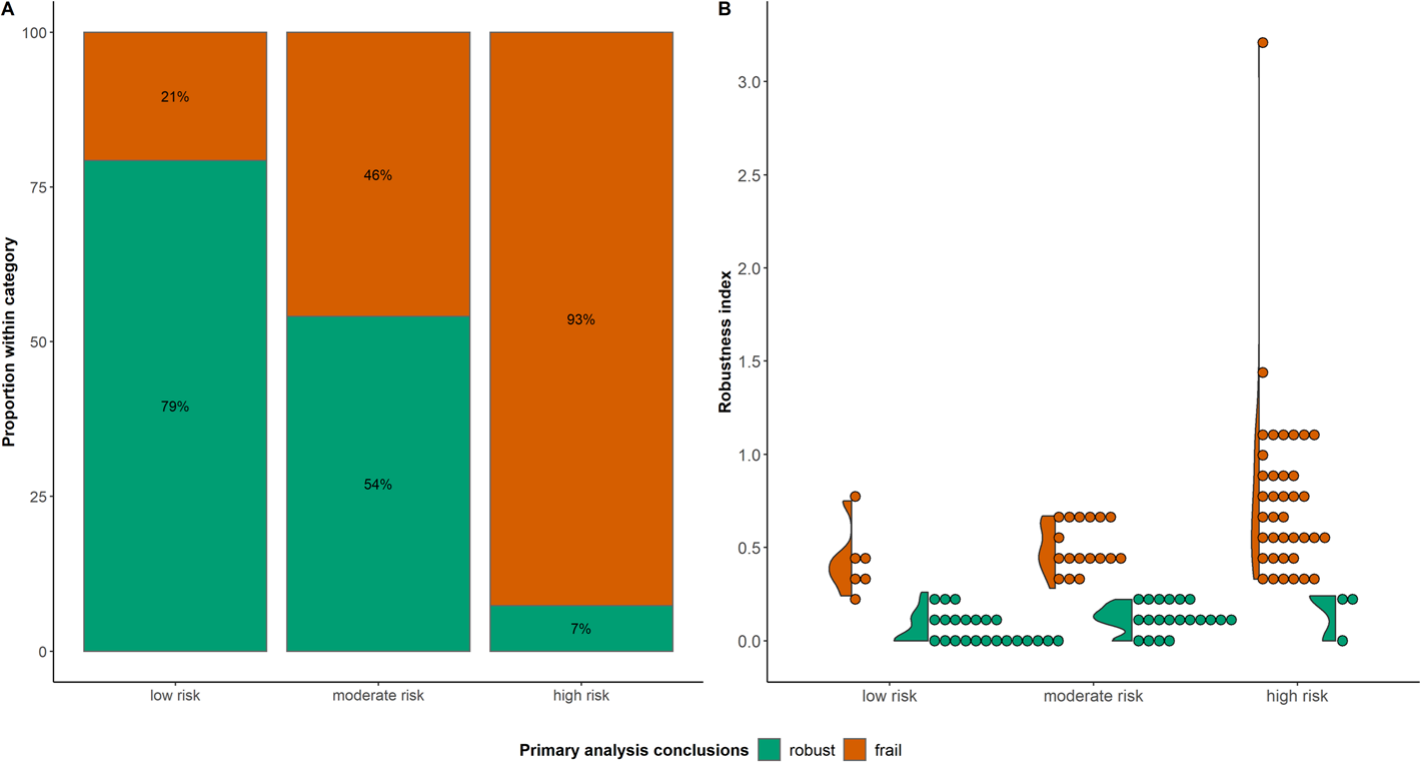
**A** Stacked barplot of the percentage of pairwise meta-analyses with robust (green bar) and frail (red bar) conclusions in each group of the *x*-axis. **B** Violin plot with integrated dots of the robustness index (RI) values of pairwise meta-analyses with robust (green colour) and frail (red colour) conclusions in each group of the *x*-axis

**Fig. 3:**
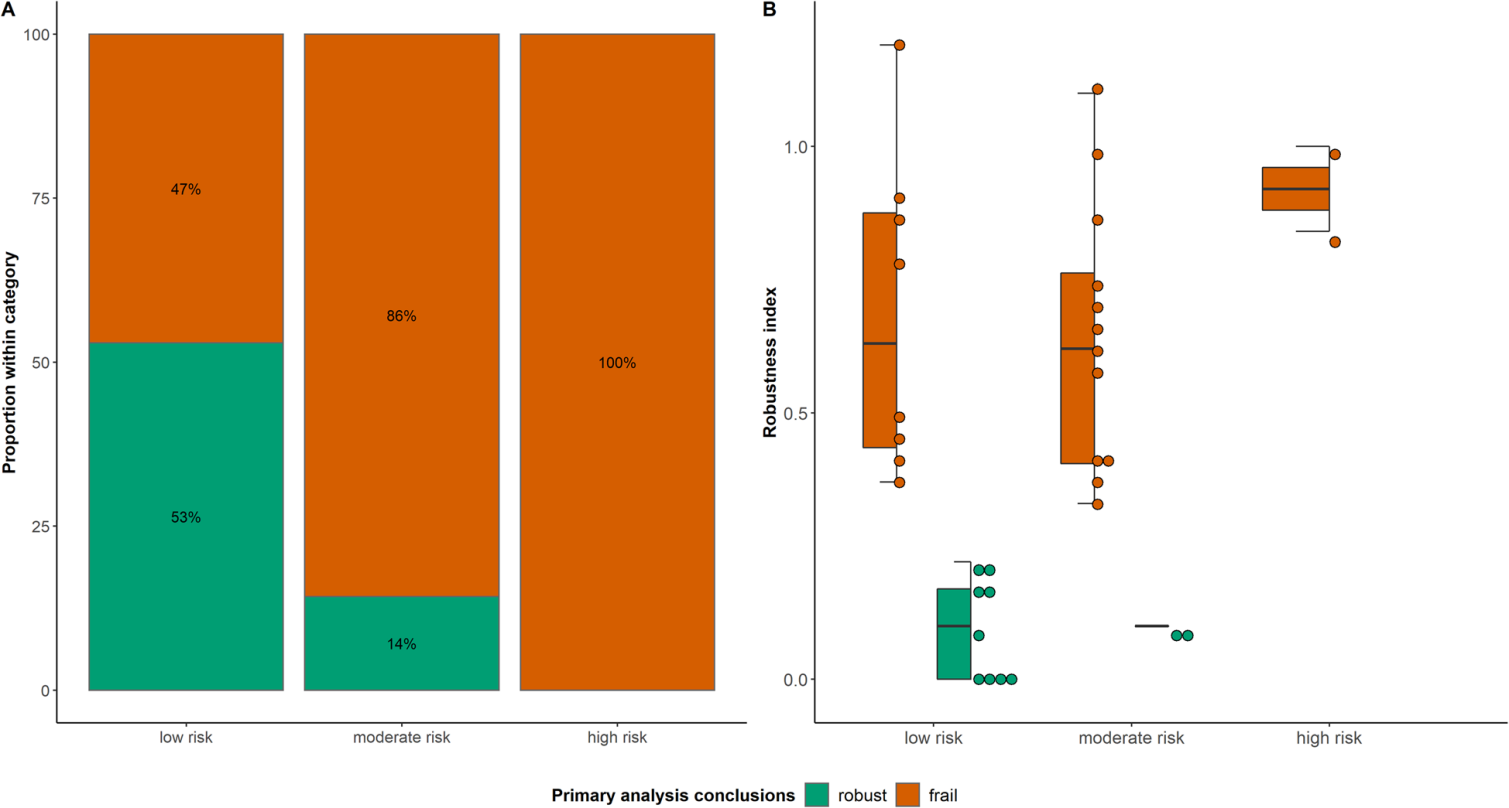
**A** Stacked barplot of the percentage of network meta-analyses with robust (green bar) and frail (red bar) conclusions in each group of the *x*-axis. **B** Boxplot with integrated dots on the maximum robustness index among the comparisons of a network with robust (green colour) and frail (red colour) conclusions in each group of the *x*-axis

Using the RI, we found that 61 (57%) PMAs and 22 (67%) NMAs failed to demonstrate robustness of the primary analysis results. The summary effect estimates of these analyses were, thus, sensitive to different assumptions about the missingness mechanisms in the compared interventions. Figures 2A and 3A depict the relative frequency of robust and frail conclusions with respect to the classification of PMAs and NMAs based on the risk of bias due to MOD in the included studies. The stacked barplots showed that the higher the risk of bias in studies, the more likely the analysis is to yield frail decisions, revealing a pattern between the risk of bias due to MOD and the credibility of findings in PMAs/NMAs. In addition, we observed frail conclusions for six PMAs and eight NMAs in the ‘low risk’ group and robust conclusions for three PMAs in the ‘high risk’ group, which may appear counterintuitive (Figs. 2A and 3A). These findings suggest that except for the case of no MOD in a PMA/NMA, the percentage of MOD in most synthesised studies may not necessarily ensure robust or frail conclusions, partially due to potential unobserved confounding. Additional file 5: Table S3 describes the characteristics of these PMAs and NMAs.

**Fig. 4:**
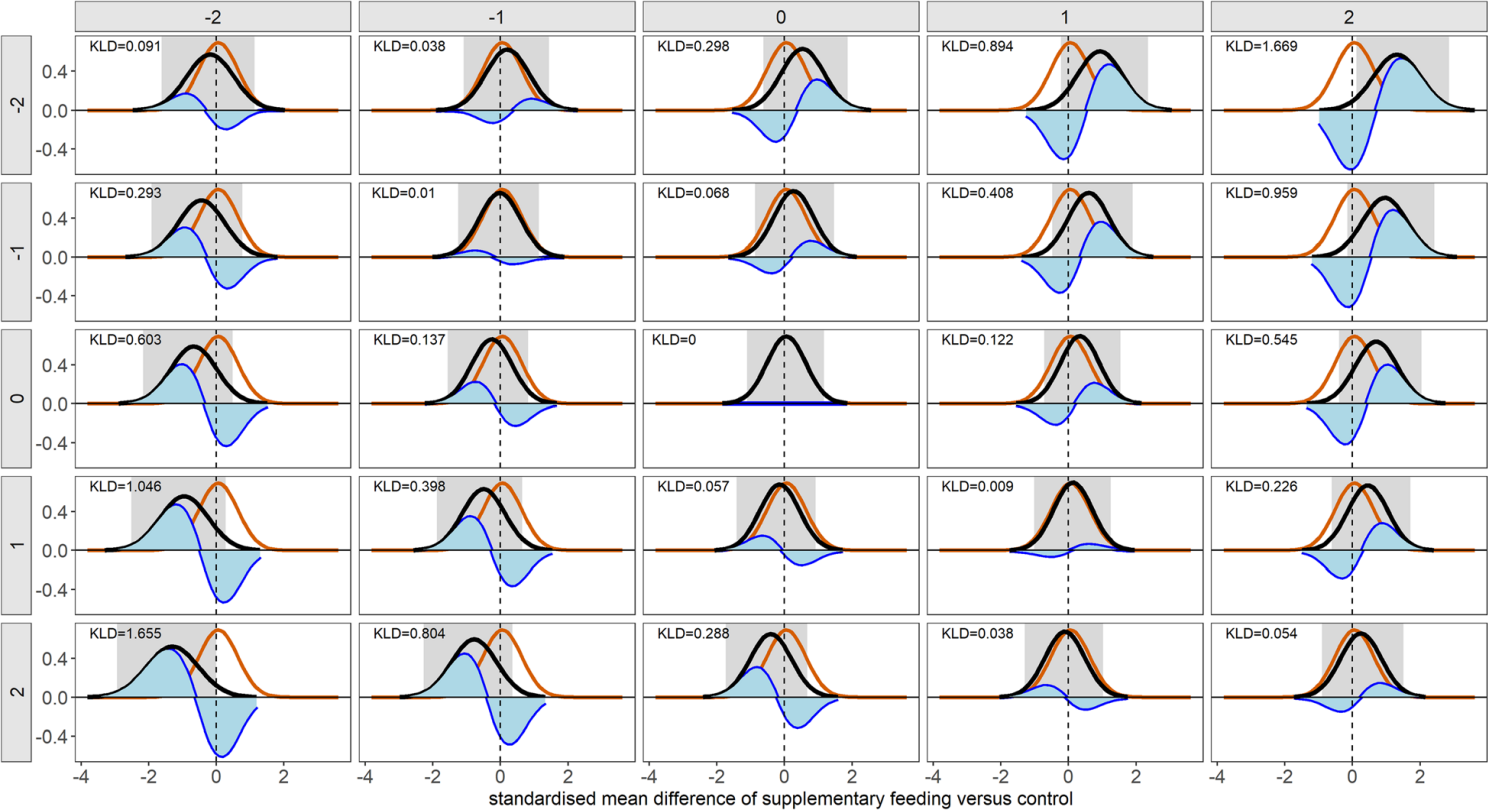
A panel of probability density plots of the summary standardised mean difference (SMD) of supplementary feeding versus the control intervention from Sguassero et al. [36]. The red line indicates the posterior distribution of the SMD under the primary analysis. The black lines indicate the posterior distribution of the SMD under the alternative re-analyses. The alternative re-analyses refer to 24 different assumptions about the informative missingness difference of means parameter in the supplementary feeding (facets at the top of the panel) and the control intervention (facets at the left of the panel). The blue area corresponds to the Kullback-Leibler divergence (KLD) measure. The vertical dotted line refers to SMD equal to zero (no difference). The grey rectangular indicates the 95% credible interval of SMD under the corresponding re-analysis

After excluding one outlying PMA, the RI had a slightly wider range of values in PMAs than in NMAs (Figs. 2B and 3B): 0 to 1.40 in PMAs and 0 to 1.31 in NMAs, where each dot represents the RI value for each of the PMA/NMA. The outlying point referred to the PMA by Sguassero et al. [36], evaluating the effect of supplementary feeding in children’s early life growth (Fig. 2B). The analysed outcome comprised four studies with 20%, 15%, 21%, and 26% total MOD, respectively. Considering the same assumptions for IMDoM in both interventions yielded the smallest Kullback-Leibler divergence measure (range 0 to 0.09) compared to the assumptions above and below the main diagonal (Fig. 4). Different IMDoM assumptions for the compared interventions affected the posterior mean of the SMD considerably, for example, IMDoM_Supplementary feeding_ = 2 and IMDoM_Control_ = − 2 (Fig. 4), yielding a striking RI of 3.22.

### Agreement with the current sensitivity analysis standards

The current sensitivity analysis standards indicated in total fewer analyses with frail conclusions than those identified by calculating and applying the threshold for the RI. Specifically, a total of 55 (39%) analyses failed to demonstrate robustness of the primary analysis results under the current sensitivity analysis standards as opposed to 83 (59%) analyses in total under the RI. For the PMAs, Cohen’s kappa statistic indicated a slight agreement between these two frameworks, though there was great uncertainty in the estimation (mean 0.19; 95% confidence interval (CI) 0.02 to 0.39). For the NMAs, Cohen’s kappa statistic indicated a fair agreement; however, the 95% CI ranged from poor to a substantial agreement (mean 0.25, 95% CI − 0.41 to 0.64).

**Fig. 5:**
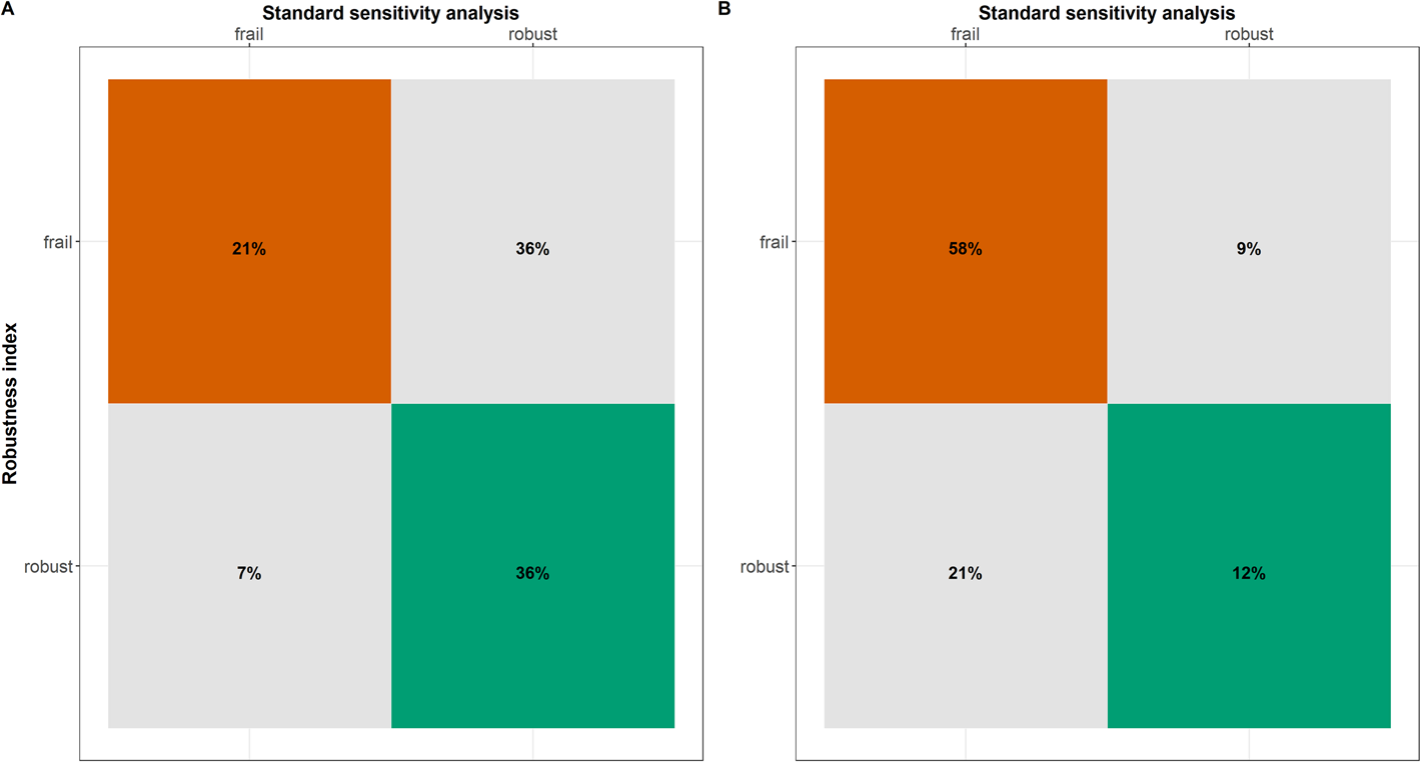
**A** Cross-tabulation of the percentage of pairwise meta-analyses with robust and frail conclusions under the robustness index and the current sensitivity analysis standards. **B** Cross-tabulation of the percentage of network meta-analyses with robust and frail conclusions under the robustness index and the current sensitivity analysis standards

For the 46 (43%) PMAs with contradictory conclusions from the compared frameworks (non-diagonal elements in Fig. 5A), we looked further into the probability density plots of the summary effects from the primary analysis and the 24 re-analyses. Seven PMAs were associated with robust conclusions based on the RI but with frail conclusions based on the current sensitivity analysis standards (Additional file 4: Figures S2 to S8) [37–43]. The statistical significance changed in at least one re-analysis using the current sensitivity analysis standards, leading to frail conclusions for these comparisons. These re-analyses referred to opposite assumptions about the missingness mechanism in the compared arms (i.e. bottom left or top right of the panels) (Additional file 4: Figures S2 to S8) [37–43]. Four of these PMAs were classified as having more studies with a low risk of bias due to MOD and the rest as having more studies with a moderate risk of bias. The same pattern was observed for the seven (21%) NMAs, where conclusions on the statistical significance changed in at least one re-analysis of the possible comparisons of the network (Fig. 5B).

In all 39 (36%) PMAs with *frail* conclusions, based on the RI, the statistical significance did not change in any re-analysis (for example, Additional file 4: Figure S9 [44]). Consequently, the current sensitivity analysis standards deduced the conclusions from these analyses to be robust. The Kullback-Leibler divergence measure was, however, systematically substantial in the opposite assumptions about the missingness mechanism in the compared arms. Therefore, the RI determined the conclusions from these analyses as frail. Almost half of these PMAs were classified as having more studies with a high risk of bias due to MOD, followed by 8 (20%) PMAs with more studies with a moderate risk of bias due to MOD. A similar pattern was observed for the three (9%) NMAs with frail conclusions under the RI but robust conclusions under the standard sensitivity analysis (Fig. 5B).

## Discussion

The primary analysis results can be sensitive to different assumptions about the missingness mechanisms in the compared interventions of the synthesised studies. The ratio of studies with low to a substantial amount of MOD can also implicate the robustness of the primary analysis results. Using the proposed RI showed almost double the number of frail conclusions compared with relying on the statistical significance of the summary effect estimate in the re-analyses. Comparing the RI with the current sensitivity analysis standards revealed that two in five analyses yielded contradictory conclusions regarding the robustness of the primary analysis results. Reliance on the statistical significance resulted in frail conclusions in analyses with a materially unaffected posterior distribution of the summary effect estimate that included the threshold for null effect in the primary and subsequent analyses. Based on the significance level of 5%, the statistical significance of these analyses changed when making more stringent assumptions.

Furthermore, the current sensitivity analysis standards yielded robust conclusions in analyses where the posterior distribution varied substantially under stringent assumptions. The statistical significance (at a 5% level) was maintained in all re-analyses of these PMAs/NMAs. The RI naturally accounted for the deviations in the location and dispersion of the posterior distribution in the re-analyses; therefore, it demonstrated the sensitivity of the primary analysis results to different assumptions.

This is the first empirical study to investigate the sensitivity of the summary effect estimates of PMAs and NMAs to different assumptions about MOD. We considered a wide range of clinically plausible assumptions about the missingness mechanisms in the compared interventions. Therefore, we were able to thoroughly investigate the sensitivity of the results to a varying degree of stringent assumptions. However, these assumptions were not tailored to the interventions and conditions under investigation. Ideally, expert opinion should be sought to determine the assumptions for the sensitivity analysis at the protocol stage of the analysis.

Furthermore, we used an objective framework to develop the robustness thresholds. These thresholds reflected the minimally allowed deviation in a general healthcare setting. Preferably, clinically specific robustness thresholds should be considered in addition to our proposed threshold.

This is also the first empirical study on systematic reviews to rely on objective criteria other than statistical significance to determine the presence or lack of robustness of the primary analysis results. Kahale et al. [8] is the most recent empirical study on the impact of MOD on the summary effect estimates from PMAs. The authors reported that only a quarter of 100 PMAs failed to demonstrate robustness based on statistical significance. Our study revealed that mere reliance on statistical significance was sensitive to the selected significance level. It, hence, declared conclusions as robust or frail in cases where the posterior distribution of the summary effect estimate differed or was materially unchanged to the different re-analyses, respectively. By employing the RI in the database of Kahale et al. [8], one may expect a higher percentage of PMAs with frail conclusions due to the substantial percentage of participants with definite or potential MOD in these PMAs (median 11.7% and interquartile range 5.6 to 23.7%).

The present study focused on the impact of two factors on the sensitivity of the primary analysis results: (1) the amount of MOD in the collated studies and (2) the different assumptions about the missingness mechanisms in the compared interventions. Potential unobserved confounding (stemming from analysing aggregate outcome data), the size and the number of the studies, and the distribution of the outcome across the studies, also constitute important factors that may affect the summary effect size, and by extent, the conclusions from a sensitivity analysis. Variability in the sample size and the distribution of the outcome should be expected and properly accounted for. In the present study, we preferred modelling the exact distribution of the binary outcome data (one-stage approach) rather than approximating the normal distribution (two-stage approach)—the latter being difficult to defend when the included studies are small, and the investigated outcome is rare [45]. Following Dias et al. [15], we have assumed approximately normally distributed sample means for the continuous outcome by convention, which may have implications for the summary SMD when the studies are small [45].

Despite the cautionary tales on the misuse of statistical significance in interpreting the study results, dichotomising the results based on a 5% significance level remains the status quo in the published literature. This study showed the merits of objectively developed decision criteria, contrary to reliance on statistical significance in isolation, to interpret the sensitivity analysis results. Therefore, we aspire for this framework to be integrated into the GRADE guidance for assessing the risk of bias due to MOD, which, coupled with plausible clinical assumptions, may uncover the comparisons and outcomes with frail conclusions [46]. In addition, the relevance and utility of our sensitivity analysis framework extend beyond the analysis of MOD. For instance, the sensitivity of the results to different prior distributions for the between-study heterogeneity parameter, different effect measures, or excluding outlying studies can be easily inferred with our proposed framework. Finally, it can be applied straightforwardly regardless of the analysis framework (frequentist or Bayesian).

An index that evaluates the consistency assumption would further help the analyst infer the degree of inconsistency in the network and whether the NMA results are valid. There are currently no recommendations to interpret the estimated inconsistency parameter as an indication of low or considerable inconsistency. Therefore, the analysts unduly rely on the statistical significance of the inconsistency parameter to infer the presence or lack of consistency.

Clinically relevant robustness thresholds would allow for contextualised conclusions regarding the robustness of the primary analysis results. For instance, deciding what constitutes a minimum clinically important difference (MCID) in the *sensitivity analysis context* could be used as the robustness threshold. Then, an RI below this threshold would signify robust primary analysis results. Preferably, the elicited threshold would be based on several experts with different experiences on the subject under investigation [47]. Then, the average of MCIDs across the experts weighted by their experience in years would comprise the robustness threshold.

## Conclusions

Interpreting the sensitivity analysis results requires objectivity and contextualisation to safeguard against spurious conclusions. The current sensitivity analysis standards rely on statistical significance; hence, they fail to fulfil these requirements. We proposed the RI as a better alternative to the current sensitivity analysis standards, which offers an objective definition of similar results and does not rely unduly on statistical significance. The RI can overhaul the current norms in applying and interpreting sensitivity analyses in systematic reviews.

## Supplementary Information

**Additional file 1.** Reference list of analysed pairwise meta-analyses from Cochrane systematic reviews.

**Additional file 2.** Reference list of analysed network meta-analyses.

**Additional file 3.** Note S1. Specification of the Bayesian models. Note S2. Exclusion due to convergence issues.

**Additional file 4.** Supplementary figures.

**Additional file 5.** Supplementary tables.

## Data Availability

The datasets generated and/or analysed during the current study are available in the https://github.com/LoukiaSpin/Empirical-Evidence-on-Robustness-in-Meta-analyses.git repository. A list of the relevant systematic reviews considered in the present study has been included in the Supplementary Information (see Additional files 1 and 2).

## Abbreviations

CI: Confidence interval
IMDoM: Informative missingness difference of means
IMOR: Informative missingness odds ratio
MAR: Missing at random
MOD: Missing participant outcome data
NMA: Network meta-analysis
OR: Odds ratio
PMA: Pairwise meta-analysis
RI: Robustness index
SMD: Standardised mean difference
