# Supplementary material for: How robust are findings of pairwise and network meta-analysis in the presence of missing participant outcome data?
Source: BMC Med. 2021 Dec 21;19:323. doi: 10.1186/s12916-021-02195-y (PMC8691029; doi:10.1186/s12916-021-02195-y)
Supplement: Supplementary file 1 — Additional file 1. Reference list of analysed pairwise meta-analyses from Cochrane systematic reviews. [file 12916_2021_2195_MOESM1_ESM.docx]

## Additional file 1

**Supplementary material for the manuscript entitled “How robust are findings of pairwise and network meta-analysis in the presence of missing participant outcome data?”**

Loukia M. Spineli^1^, Chrysostomos Kalyvas^2^, Katerina Papadimitropoulou^3,4^

^1^Midwifery Research and Education Unit, Hannover Medical School, Hannover, Germany

^2^Biostatistics and Research Decision Sciences, MSD Europe Inc., Brussels, Belgium

^3^Clinical Epidemiology, Leiden University Medical Center, Leiden, The Netherlands

^4^Data Science and Biometrics, Danone Nutricia Research, Utrecht, The Netherlands

## Reference list of analysed pairwise meta-analyses from Cochrane systematic reviews^[[1]](#footnote-1)^

**Binary outcome**

***Depression, Anxiety and Neurosis Group***

1. van Marwijk H, van Marwijk H, Allick G, Wegman F, Bax A, Riphagen II. Alprazolam for depression. Cochrane Database Syst Rev. 2012; (7): CD007139. doi: 10.1002/14651858.CD007139.pub2.
2. Guaiana G, Barbui C, Hotopf M. Amitriptyline for depression. Cochrane Database Syst Rev. 2007; (3): CD004186. doi: 10.1002/14651858.CD004186.pub2.
3. Leucht C, Huhn M, Leucht S. Amitriptyline versus placebo for major depressive disorder. Cochrane Database Syst Rev. 2012; (12): CD009138. doi: 10.1002/14651858.CD009138.pub2.
4. Mottram PG, Wilson K, Strobl JJ. Antidepressants for depressed elderly. Cochrane Database Syst Rev. 2006; (1): CD003491. doi: 10.1002/14651858.CD003491.pub2.
5. Rayner L, Price A, Evans A, Valsraj K, Higginson IJ, Hotopf M. Antidepressants for depression in physically ill people. Cochrane Database Syst Rev. 2010; (3): CD007503. doi: 10.1002/14651858.CD007503.pub2.
6. Kapczinski FFK, Silva de Lima M, dos Santos Souza JJSS, Batista Miralha da Cunha AABC, Schmitt RRS. Antidepressants for generalized anxiety disorder. Cochrane Database Syst Rev. 2003; (2): CD003592. doi: 10.1002/14651858.CD003592.
7. Furukawa TA, Streiner D, Young LT, Kinoshita Y. Antidepressants plus benzodiazepines for major depression. Cochrane Database Syst Rev. 2001; (3): CD001026. doi: 10.1002/14651858.CD001026.
8. Arroll B, Elley CR, Fishman T, Goodyear-Smith FA, Kenealy T, Blashki G, Kerse N, MacGillivray S. Antidepressants versus placebo for depression in primary care. Cochrane Database Syst Rev. 2009; (3): CD007954. doi: 10.1002/14651858.CD007954.
9. Bacaltchuk J, Hay PPJ. Antidepressants versus placebo for people with bulimia nervosa. Cochrane Database Syst Rev. 2003; (4): CD003391. doi: 10.1002/14651858.CD003391.
10. Hay PPJ, Claudino AM, Kaio MH. Antidepressants versus psychological treatments and their combination for bulimia nervosa. Cochrane Database Syst Rev. 2001; (4): CD003385. doi: 10.1002/14651858.CD003385.
11. Cipriani A, Purgato M, Furukawa TA, Trespidi C, Imperadore G, Signoretti A, Churchill R, Watanabe N, Barbui C. Citalopram versus other anti-depressive agents for depression. Cochrane Database Syst Rev. 2012; (7): CD006534. doi: 10.1002/14651858.CD006534.pub2.
12. James AACJ, Soler A, Weatherall RRW. Cognitive behavioural therapy for anxiety disorders in children and adolescents. Cochrane Database Syst Rev. 2005; (4): CD004690. doi: 10.1002/14651858.CD004690.pub2.
13. Furukawa TA, Watanabe N, Churchill R. Combined psychotherapy plus antidepressants for panic disorder with or without agoraphobia. Cochrane Database Syst Rev. 2007; (1): CD004364. doi: 10.1002/14651858.CD004364.pub2.
14. Silva de Lima M, Moncrieff J, Soares B. Drugs versus placebo for dysthymia. Cochrane Database Syst Rev. 2005; (2): CD001130. doi: 10.1002/14651858.CD001130.
15. Cipriani A, Koesters M, Furukawa TA, Nosè M, Purgato M, Omori IM, Trespidi C, Barbui C. Duloxetine versus other anti-depressive agents for depression. Cochrane Database Syst Rev. 2012; (10): CD006533. doi: 10.1002/14651858.CD006533.pub2.
16. Cipriani A, Santilli C, Furukawa TA, Signoretti A, Nakagawa A, McGuire H, Churchill R, Barbui C. Escitalopram versus other antidepressive agents for depression. Cochrane Database Syst Rev. 2009; (2): CD006532. doi: 10.1002/14651858.CD006532.pub2.
17. Fisher CA, Hetrick SE, Rushford N. Family therapy for anorexia nervosa. Cochrane Database Syst Rev. 2010; (4): CD004780. doi: 10.1002/14651858.CD004780.pub2.
18. Cipriani A, Brambilla P, Furukawa TA, Geddes J, Gregis M, Hotopf M, Malvini L, Barbui C. Fluoxetine versus other types of pharmacotherapy for depression. Cochrane Database Syst Rev. 2005; (4): CD004185. doi: 10.1002/14651858.CD004185.pub2.
19. Omori IM, Watanabe N, Nakagawa A, Cipriani A, Barbui C, McGuire H, Churchill R, Furukawa TA. Fluvoxamine versus other anti-depressive agents for depression. Cochrane Database Syst Rev. 2010; (3): CD006114. doi: 10.1002/14651858.CD006114.pub2.
20. Guaiana G, Barbui C, Cipriani A. Hydroxyzine for generalised anxiety disorder. Cochrane Database Syst Rev. 2010; (12): CD006815. doi: 10.1002/14651858.CD006815.pub2.
21. Tuunainen A, Kripke DF, Endo T. Light therapy for non-seasonal depression. Cochrane Database Syst Rev. 2004; (2): CD004050. doi: 10.1002/14651858.CD004050.pub2.
22. Burgess SSA, Geddes J, Hawton KKE, Taylor MJ, Townsend E, Jamison K, Goodwin G. Lithium for maintenance treatment of mood disorders. Cochrane Database Syst Rev. 2001; (3): CD003013. doi: 10.1002/14651858.CD003013.
23. Cipriani A, Smith KA, Burgess SSA, Carney SM, Goodwin G, Geddes J. Lithium versus antidepressants in the long-term treatment of unipolar affective disorder. Cochrane Database Syst Rev. 2006; (4): CD003492. doi: 10.1002/14651858.CD003492.pub2.
24. Furukawa TA, McGuire H, Barbui C. Low dosage tricyclic antidepressants for depression. Cochrane Database Syst Rev. 2003; (3): CD003197. doi: 10.1002/14651858.CD003197.
25. Watanabe N, Omori IM, Nakagawa A, Cipriani A, Barbui C, Churchill R, Furukawa TA. Mirtazapine versus other antidepressive agents for depression. Cochrane Database Syst Rev. 2011; (12): CD006528. doi: 10.1002/14651858.CD006528.pub2.
26. Hetrick SE, McKenzie JE, Cox GR, Simmons MB, Merry SN. Newer generation antidepressants for depressive disorders in children and adolescents. Cochrane Database Syst Rev. 2012; (11): CD004851. doi: 10.1002/14651858.CD004851.pub3.
27. Wijkstra J, Lijmer J, Balk F, Geddes J, Nolen WA. Pharmacological treatment for psychotic depression. Cochrane Database Syst Rev. 2005; (4): CD004044. doi: 10.1002/14651858.CD004044.pub2.
28. Ipser JC, Carey P, Dhansay Y, Fakier N, Seedat S, Stein DJ. Pharmacotherapy augmentation strategies in treatment-resistant anxiety disorders. Cochrane Database Syst Rev. 2006; (4): CD005473. doi: 10.1002/14651858.CD005473.pub2.
29. Ipser JC, Stein DJ, Hawkridge S, Hoppe L. Pharmacotherapy for anxiety disorders in children and adolescents. Cochrane Database Syst Rev. 2009; (3): CD005170. doi: 10.1002/14651858.CD005170.pub2.
30. Stein DJ, Ipser JC, Seedat S. Pharmacotherapy for post traumatic stress disorder (PTSD). Cochrane Database Syst Rev. 2006; (1): CD002795. doi: 10.1002/14651858.CD002795.pub2.
31. Rose SC, Bisson J, Churchill R, Wessely S. Psychological debriefing for preventing post traumatic stress disorder (PTSD). Cochrane Database Syst Rev. 2002; (2): CD000560. doi: 10.1002/14651858.CD000560.
32. Hunot V, Churchill R, Teixeira V, Silva de Lima M. Psychological therapies for generalised anxiety disorder. Cochrane Database Syst Rev. 2007; (1): CD001848. doi: 10.1002/14651858.CD001848.pub4.
33. Cox GR, Callahan P, Churchill R, Hunot V, Merry SN, Parker AG, Hetrick SE. Psychological therapies versus antidepressant medication, alone and in combination for depression in children and adolescents. Cochrane Database Syst Rev. 2012; (11): CD008324. doi: 10.1002/14651858.CD008324.pub2.
34. Candy B, Jones L, Williams R, Tookman A, King M. Psychostimulants for depression. Cochrane Database Syst Rev. 2008; (2): CD006722. doi: 10.1002/14651858.CD006722.pub2.
35. Depping AM, Komossa K, Kissling W, Leucht S. Second-generation antipsychotics for anxiety disorders. Cochrane Database Syst Rev. 2010; (12): CD008120. doi: 10.1002/14651858.CD008120.pub2.
36. Komossa K, Depping AM, Gaudchau A, Kissling W, Leucht S. Second-generation antipsychotics for major depressive disorder and dysthymia. Cochrane Database Syst Rev. 2010; (12): CD008121. doi: 10.1002/14651858.CD008121.pub2.
37. Soomro GM, Altman DG, Rajagopal S, Oakley Browne M. Selective serotonin re-uptake inhibitors (SSRIs) versus placebo for obsessive compulsive disorder (OCD). Cochrane Database Syst Rev. 2008; (1): CD001765. doi: 10.1002/14651858.CD001765.pub3.
38. Cipriani A, La Ferla T, Furukawa TA, Signoretti A, Nakagawa A, Churchill R, McGuire H, Barbui C. Sertraline versus other antidepressive agents for depression. Cochrane Database Syst Rev. 2010; (4): CD006117. doi: 10.1002/14651858.CD006117.pub4.
39. Linde K, Berner MM, Kriston L. St John’s wort for major depression. Cochrane Database Syst Rev. 2008; (4): CD000448. doi: 10.1002/14651858.CD000448.pub3.
40. Hazell P, O’Connell D, Heathcote D, Henry DA. Tricyclic drugs for depression in children and adolescents. Cochrane Database Syst Rev. 2002; (2): CD002317. doi: 10.1002/14651858.CD002317.

***Schizophrenia Group***

1. Silveira da Mota Neto JI, Soares BGO, Silva de Lima M. Amisulpride for schizophrenia. Cochrane Database Syst Rev. 2002; (2): CD001357. doi: 10.1002/14651858.CD001357.
2. El-Sayeh HGG, Morganti C. Aripiprazole for schizophrenia. Cochrane Database Syst Rev. 2006; (2): CD004578. doi: 10.1002/14651858.CD004578.pub3.
3. Bhattacharjee J, El-Sayeh HGG. Aripiprazole versus typical antipsychotic drugs for schizophrenia. Cochrane Database Syst Rev. 2008; (3): CD006617. doi: 10.1002/14651858.CD006617.pub3.
4. Gillies D, Beck A, McCloud A, Rathbone J. Benzodiazepines for psychosis-induced aggression or agitation. Cochrane Database Syst Rev. 2005; (4): CD003079. doi: 10.1002/14651858.CD003079.pub2.
5. Dold M, Li C, Tardy M, Khorsand V, Gillies D, Leucht S. Benzodiazepines for schizophrenia. Cochrane Database Syst Rev. 2012; (11): CD006391. doi: 10.1002/14651858.CD006391.pub2.
6. Leucht S, Kissling W, McGrath J, White P. Carbamazepine for schizophrenia. Cochrane Database Syst Rev. 2007; (3): CD001258. doi: 10.1002/14651858.CD001258.pub2.
7. Almerie MQ, Alkhateeb H, Essali A, Matar HE, Rezk E. Cessation of medication for people with schizophrenia already stable on chlorpromazine. Cochrane Database Syst Rev. 2007; (1): CD006329. doi: 10.1002/14651858.CD006329.
8. Adams CE, Awad G, Rathbone J, Thornley B. Chlorpromazine versus placebo for schizophrenia. Cochrane Database Syst Rev. 2007; (2): CD000284. doi: 10.1002/14651858.CD000284.pub2.
9. Tammenmaa I, McGrath J, Sailas EES, Soares-Weiser K. Cholinergic medication for neuroleptic-induced tardive dyskinesia. Cochrane Database Syst Rev. 2002; (3): CD000207. doi: 10.1002/14651858.CD000207.
10. Berk M, Rathbone J, Mandriota-Carpenter SL. Clotiapine for acute psychotic illnesses. Cochrane Database Syst Rev. 2004; (4): CD002304. doi: 10.1002/14651858.CD002304.pub2.
11. Essali A, Al-Haj Haasan N, Li C, Rathbone J. Clozapine versus typical neuroleptic medication for schizophrenia. Cochrane Database Syst Rev. 2009; (1): CD000059. doi: 10.1002/14651858.CD000059.pub2.
12. Jones C, Hacker D, Cormac I, Meaden A, Irving CB. Cognitive behaviour therapy versus other psychosocial treatments for schizophrenia. Cochrane Database Syst Rev. 2012; (4): CD008712. doi: 10.1002/14651858.CD008712.pub2.
13. Murphy S, Irving CB, Adams CE, Driver R. Crisis intervention for people with severe mental illnesses. Cochrane Database Syst Rev. 2012; (5): CD001087. doi: 10.1002/14651858.CD001087.pub4.
14. Marshall M, Crowther R, Sledge WH, Rathbone J, Soares-Weiser K. Day hospital versus admission for acute psychiatric disorders. Cochrane Database Syst Rev. 2011; (12): CD004026. doi: 10.1002/14651858.CD004026.pub2.
15. David A, Adams CE, Quraishi SN. Depot flupenthixol decanoate for schizophrenia or other similar psychotic disorders. Cochrane Database Syst Rev. 1999; (2): CD001470. doi: 10.1002/14651858.CD001470.
16. Abhijnhan A, Adams CE, David A, Ozbilen M. Depot fluspirilene for schizophrenia. Cochrane Database Syst Rev. 2007; (1): CD001718. doi: 10.1002/14651858.CD001718.pub2.
17. Quraishi SN, David A, Brasil MA, Alheira FV. Depot haloperidol decanoate for schizophrenia. Cochrane Database Syst Rev. 1999; (1): CD001361. doi: 10.1002/14651858.CD001361.
18. Dinesh M, David A, Quraishi SN. Depot pipotiazine palmitate and undecylenate for schizophrenia Cochrane Database Syst Rev. 2004; (3): CD001720. doi: 10.1002/14651858.CD001720.pub2.
19. Tharyan P, Adams CE. Electroconvulsive therapy for schizophrenia. Cochrane Database Syst Rev. 2005; (2): CD000076. doi: 10.1002/14651858.CD000076.pub2.
20. Pharoah F, Mari JJ, Rathbone J, Wong W. Family intervention for schizophrenia. Cochrane Database Syst Rev. 2010; (12): CD000088. doi: 10.1002/14651858.CD000088.pub3.
21. Alabed S, Latifeh Y, Mohammad HA, Rifai A. Gamma-aminobutyric acid agonists for neuroleptic-induced tardive dyskinesia. Cochrane Database Syst Rev. 2011; (4): CD000203. doi: 10.1002/14651858.CD000203.pub3.
22. Tiihonen J, Wahlbeck K. Glutamatergic drugs for schizophrenia. Cochrane Database Syst Rev. 2006; (2): CD003730. doi: 10.1002/14651858.CD003730.pub2.
23. Donnelly L, Waraich PS, Adams CE, Hamill KM, Marti J, Roqué i Figuls M, Rathbone J. Haloperidol dose for the acute phase of schizophrenia. Cochrane Database Syst Rev. 2002; (2): CD001951. doi: 10.1002/14651858.CD001951.
24. Powney MJ, Adams CE, Jones H. Haloperidol for psychosis-induced aggression or agitation (rapid tranquillisation). Cochrane Database Syst Rev. 2012; (11): CD009377. doi: 10.1002/14651858.CD009377.pub2.
25. Leucht C, Kitzmantel M, Kane J, Leucht S, Chua WLLC. Haloperidol versus chlorpromazine for schizophrenia. Cochrane Database Syst Rev. 2008; (1): CD004278. doi: 10.1002/14651858.CD004278.pub2.
26. Irving CB, Adams CE, Lawrie S. Haloperidol versus placebo for schizophrenia. Cochrane Database Syst Rev. 2006; (4): CD003082. doi: 10.1002/14651858.CD003082.pub2.
27. Dieterich M, Irving CB, Park B, Marshall M. Intensive case management for severe mental illness. Cochrane Database Syst Rev. 2010; (10): CD007906. doi: 10.1002/14651858.CD007906.pub2.
28. Roberts L, Ahmed I, Hall S, Davison A. Intercessory prayer for the alleviation of ill health. Cochrane Database Syst Rev. 2009; (2): CD000368. doi: 10.1002/14651858.CD000368.pub3.
29. Alwan N, Johnstone P, Zolese G. Length of hospitalisation for people with severe mental illness. Cochrane Database Syst Rev. 2008; (1): CD000384. doi: 10.1002/14651858.CD000384.pub2.
30. Leucht S, Kissling W, McGrath J. Lithium for schizophrenia. Cochrane Database Syst Rev. 2007; (3): CD003834. doi: 10.1002/14651858.CD003834.pub2.
31. Chakrabarti A, Bagnall AM, Chue P, Fenton M, Palaniswamy V, Wong W, Xia J. Loxapine for schizophrenia. Cochrane Database Syst Rev. 2007; (4): CD001943. doi: 10.1002/14651858.CD001943.pub2.
32. Leucht S, Tardy M, Komossa K, Heres S, Kissling W, Davis JM. Maintenance treatment with antipsychotic drugs for schizophrenia. Cochrane Database Syst Rev. 2012; (5): CD008016. doi: 10.1002/14651858.CD008016.pub2.
33. Tuunainen A, Wahlbeck K. Newer atypical antipsychotic medication versus clozapine for schizophrenia. Cochrane Database Syst Rev. 2000; (2): CD000966. doi: 10.1002/14651858.CD000966.
34. Duggan L, Fenton M, Rathbone J, Dardennes R, El-Dosoky A, Indran S. Olanzapine for schizophrenia. Cochrane Database Syst Rev. 2005; (2): CD001359. doi: 10.1002/14651858.CD001359.pub2.
35. Komossa K, Rummel-Kluge C, Hunger H, Schmid F, Schwarz S, Duggan L, Kissling W, Leucht S. Olanzapine versus other atypical antipsychotics for schizophrenia. Cochrane Database Syst Rev. 2010; (3): CD006654. doi: 10.1002/14651858.CD006654.pub2.
36. Nussbaum AM, Stroup TS. Oral paliperidone for schizophrenia. Cochrane Database Syst Rev. 2008; (2): CD006369. doi: 10.1002/14651858.CD006369.pub2.
37. Kumar A, Strech D. Zuclopenthixol dihydrochloride for schizophrenia. Cochrane Database Syst Rev. 2005; (4): CD005474. doi: 10.1002/14651858.CD005474.
38. da Silva Freire Coutinho E, Fenton M, Quraishi SN. Zuclopenthixol decanoate for schizophrenia and other serious mental illnesses. Cochrane Database Syst Rev. 1999; (3): CD001164. doi: 10.1002/14651858.CD001164.
39. Jayakody K, Gibson RC, Kumar A, Gunadasa S. Zuclopenthixol acetate for acute schizophrenia and similar serious mental illnesses. Cochrane Database Syst Rev. 2012; (4): CD000525. doi: 10.1002/14651858.CD000525.pub3.
40. DeSilva P, Fenton M, Rathbone J. Zotepine for schizophrenia. Cochrane Database Syst Rev. 2006; (4): CD001948. doi: 10.1002/14651858.CD001948.pub2.
41. Marques LDO, Soares B, Silva de Lima M. Trifluoperazine for schizophrenia. Cochrane Database Syst Rev. 2004; (1): CD003545. doi: 10.1002/14651858.CD003545.pub2.
42. Buckley LA, Pettit TACL, Adams CE. Supportive therapy for schizophrenia. Cochrane Database Syst Rev. 2007; (3): CD004716. doi: 10.1002/14651858.CD004716.pub3.
43. Soares BGO, Fenton M, Chue P. Sulpiride for schizophrenia. Cochrane Database Syst Rev. 1999; (1): CD001162. doi: 10.1002/14651858.CD001162.
44. Hunter R, Kennedy E, Song F, Gadon L, Irving CB. Risperidone versus typical antipsychotic medication for schizophrenia. Cochrane Database Syst Rev. 2003; (2): CD000440. doi: 10.1002/14651858.CD000440.
45. Rattehalli RD, Jayaram MB, Smith M. Risperidone versus placebo for schizophrenia. Cochrane Database Syst Rev. 2010; (1): CD006918. doi: 10.1002/14651858.CD006918.pub2.
46. Komossa K, Rummel-Kluge C, Schwarz S, Schmid F, Hunger H, Kissling W, Leucht S. Risperidone versus other atypical antipsychotics for schizophrenia. Cochrane Database Syst Rev. 2011; (1): CD006626. doi: 10.1002/14651858.CD006626.pub2.
47. Gilbody S, Bagnall AM, Duggan L, Tuunainen A. Risperidone versus other atypical antipsychotic medication for schizophrenia. Cochrane Database Syst Rev. 2010; (3): CD002306. doi: 10.1002/14651858.CD002306.
48. Jayaram MB, Hosalli P, Stroup TS. Risperidone versus olanzapine for schizophrenia. Cochrane Database Syst Rev. 2010; (2): CD005237. doi: 10.1002/14651858.CD005237.pub2.
49. Li C, Xia J, Wang J. Risperidone dose for schizophrenia. Cochrane Database Syst Rev. 2012; (4): CD007474. doi: 10.1002/14651858.CD007474.pub2.
50. Rathbone J, McMonagle T. Pimozide for schizophrenia or related psychoses. Cochrane Database Syst Rev. 2007; (3): CD001949. doi: 10.1002/14651858.CD001949.pub2.
51. Hartung B, Wada M, Laux G, Leucht S. Perphenazine for schizophrenia. Cochrane Database Syst Rev. 2005; (1): CD003443. doi: 10.1002/14651858.CD003443.pub2.
52. Nussbaum AM, Stroup TS. Paliperidone palmitate for schizophrenia. Cochrane Database Syst Rev. 2012; (6): CD008296. doi: 10.1002/14651858.CD008296.pub2.

***Developmental, Psychosocial and Learning Problems Group***

1. Armelius BÅ, Andreassen TH. Cognitive-behavioral treatment for antisocial behavior in youth in residential treatment. Cochrane Database Syst Rev. 2007; (4): CD005650. doi: 10.1002/14651858.CD005650.pub2.
2. Dobson D, Lucassen PLBJ, Miller JJ, Vlieger AM, Prescott P, Lewith G. Manipulative therapies for infantile colic. Cochrane Database Syst Rev. 2012; (12): CD004796. doi: 10.1002/14651858.CD004796.pub2.
3. Jesner OS, Aref-Adib M, Coren E. Risperidone for autism spectrum disorder. Cochrane Database Syst Rev. 2007; (1): CD005040. doi: 10.1002/14651858.CD005040.pub2.

**Continuous outcome**

***Depression, Anxiety and Neurosis Group***

1. Roberts NP, Kitchiner NJ, Kenardy J, Bisson JI. Early psychological interventions to treat acute traumatic stress symptoms. Cochrane Database Syst Rev. 2010; (3): CD007944. doi: 10.1002/14651858.CD007944.pub2.
2. Rimer J, Dwan K, Lawlor DA, Greig CA, McMurdo M, Morley W, Mead GE. Exercise for depression. Cochrane Database Syst Rev. 2012; (7): CD004366. doi: 10.1002/14651858.CD004366.pub5.
3. Edmonds M, McGuire H, Price JR. Exercise therapy for chronic fatigue syndrome. Cochrane Database Syst Rev. 2004; (3): CD003200. doi: 10.1002/14651858.CD003200.pub2.
4. Taylor MJ, Wilder H, Bhagwagar Z, Geddes J. Inositol for depressive disorders. Cochrane Database Syst Rev. 2004; (1): CD004049. doi: 10.1002/14651858.CD004049.pub2.
5. Arends I, Bruinvels DJ, Rebergen DS, Nieuwenhuijsen K, Madan I, Neumeyer-Gromen A, Bültmann U, Verbeek JH. Interventions to facilitate return to work in adults with adjustment disorders. Cochrane Database Syst Rev. 2012; (12): CD006389. doi: 10.1002/14651858.CD006389.pub2.
6. Ipser JC, Carey P, Dhansay Y, Fakier N, Seedat S, Stein DJ. Pharmacotherapy augmentation strategies in treatment-resistant anxiety disorders. Cochrane Database Syst Rev. 2006; (4): CD005473. doi: 10.1002/14651858.CD005473.pub2.
7. Gava I, Barbui C, Aguglia E, Carlino D, Churchill R, De Vanna M, McGuire H. Psychological treatments versus treatment as usual for obsessive compulsive disorder (OCD). Cochrane Database Syst Rev. 2007; (2): CD005333. doi: 10.1002/14651858.CD005333.pub2.
8. Wilson K, Mottram PG, Vassilas C. Psychotherapeutic treatments for older depressed people. Cochrane Database Syst Rev. 2008; (1): CD004853. doi: 10.1002/14651858.CD004853.pub2.

***Schizophrenia Group***

1. Kennedy E, Kumar A, Datta SS. Antipsychotic medication for childhood-onset schizophrenia. Cochrane Database Syst Rev. 2007; (3): CD004027. doi: 10.1002/14651858.CD004027.pub2.
2. Schwarz C, Volz A, Li C, Leucht S. Valproate for schizophrenia. Cochrane Database Syst Rev. 2008; (3): CD004028. doi: 10.1002/14651858.CD004028.pub3.

***Developmental, Psychosocial and Learning Problems Group***

1. Sguassero Y, de Onis M, Bonotti AM, Carroli G. Community-based supplementary feeding for promoting the growth of children under five years of age in low and middle income countries. Cochrane Database Syst Rev. 2012; (6): CD005039. doi: 10.1002/14651858.CD005039.pub3.
2. Law J, Garrett Z, Nye C. Speech and language therapy interventions for children with primary speech and language delay or disorder. Cochrane Database Syst Rev. 2003; (3): CD004110. doi: 10.1002/14651858.CD004110.
3. Macdonald G, Higgins JPT, Ramchandani P, Valentine JC, Bronger LP, Klein P, O’Daniel R, Pickering M, Rademaker B, Richardson G, Taylor M. Cognitive-behavioural interventions for children who have been sexually abused. Cochrane Database Syst Rev. 2012; (5): CD001930. doi: 10.1002/14651858.CD001930.pub3.

1. Reviews published before 2009 have been edited. Citation is as suggested within the reviews. [↑](#footnote-ref-1)
