# Supplementary material for: How robust are findings of pairwise and network meta-analysis in the presence of missing participant outcome data?
Source: BMC Med. 2021 Dec 21;19:323. doi: 10.1186/s12916-021-02195-y (PMC8691029; doi:10.1186/s12916-021-02195-y)
Supplement: Supplementary file 2 — Additional file 2. Reference list of analysed network meta-analyses. [file 12916_2021_2195_MOESM2_ESM.docx]

**Additional file 2**

**Supplementary material for the manuscript entitled “How robust are findings of pairwise and network meta-analysis in the presence of missing participant outcome data?”**

Loukia M. Spineli^1^, Chrysostomos Kalyvas^2^, Katerina Papadimitropoulou^3,4^

^1^Midwifery Research and Education Unit, Hannover Medical School, Hannover, Germany

^2^Biostatistics and Research Decision Sciences, MSD Europe Inc., Brussels, Belgium

^3^Clinical Epidemiology, Leiden University Medical Center, Leiden, The Netherlands

^4^Data Science and Biometrics, Danone Nutricia Research, Utrecht, The Netherlands

## Reference list of analysed network meta-analyses

**Binary outcome**

1. Cipriani A, Furukawa TA, Salanti G, Geddes JR, Higgins JP, Churchill R, et al. Comparative efficacy and acceptability of 12 new-generation antidepressants: a multiple-treatments meta-analysis. Lancet. 2009; 373: 746-58.
2. Edwards SJ, Clarke MJ, Wordsworth S, Welton NJ. Carbapenems versus other beta-lactams in the treatment of hospitalised patients with infection: a mixed treatment comparison. Curr Med Res Opin. 2009; 25: 251-61.
3. Baker WL, Baker EL, Coleman CI. Pharmacologic treatments for chronic obstructive pulmonary disease: a mixed-treatment comparison meta-analysis. Pharmacotherapy. 2009; 29: 891-905.
4. Burch J, Paulden M, Conti S, Stock C, Corbett M, Welton NJ, et al. Antiviral drugs for the treatment of influenza: a systematic review and economic evaluation. Health Technol Assess. 2009; 13: 1-265, iii-iv.
5. Uthman OA, Abdulmalik J. Comparative efficacy and acceptability of pharmacotherapeutic agents for anxiety disorders in children and adolescents: a mixed treatment comparison meta-analysis. Curr Med Res Opin. 2010; 26: 53-9.
6. Bottomley JM, Taylor RS, Ryttov J. The effectiveness of two-compound formulation calcipotriol and betamethasone dipropionate gel in the treatment of moderately severe scalp psoriasis: a systematic review of direct and indirect evidence. Curr Med Res Opin. 2011; 27: 251-68.
7. Costa J, Fareleira F, Ascenção R, Borges M, Sampaio C, Vaz-Carneiro A. Clinical comparability of the new antiepileptic drugs in refractory partial epilepsy: a systematic review and meta-analysis. Epilepsia. 2011; 52: 1280-91.
8. Makani H, Bangalore S, Romero J, Wever-Pinzon O, Messerli FH. Effect of renin-angiotensin system blockade on calcium channel blocker-associated peripheral edema. Am J Med. 2011; 124: 128-35.
9. Virgili G, Novielli N, Menchini F, Murro V, Giacomelli G. Pharmacological treatments for neovascular age-related macular degeneration: can mixed treatment comparison meta-analysis be useful? Curr Drug Targets. 2011; 12: 212-20.
10. Gallego-Galisteo M, Villa-Rubio A, Alegre-del Rey E, Márquez-Fernández E, Ramos-Báez JJ. Indirect comparison of biological treatments in refractory rheumatoid arthritis. J Clin Pharm Ther. 2012; 37: 301-7.
11. Filippini G, Del Giovane C, Vacchi L, D'Amico R, Di Pietrantonj C, Beecher D, et al. Immunomodulators and immunosuppressants for multiple sclerosis: a network meta-analysis. Cochrane Database Syst Rev. 2013; (6): CD008933.
12. Gao L, Xia L, Zhao FL, Li SC. Clinical efficacy and safety of the newer antiepileptic drugs as adjunctive treatment in adults with refractory partial-onset epilepsy: a meta-analysis of randomized placebo-controlled trials. Epilepsy Res. 2013; 103: 31-44.
13. Khan N, Shah D, Tongbram V, Verdian L, Hawkins N. The efficacy and tolerability of perampanel and other recently approved anti-epileptic drugs for the treatment of refractory partial onset seizure: a systematic review and Bayesian network meta-analysis. Curr Med Res Opin. 2013; 29: 1001-13.
14. Liu J, Dong J, Wang L, Su Y, Yan P, Sun S. Comparative efficacy and acceptability of antidepressants in Parkinson's disease: a network meta-analysis. PLoS One. 2013; 8: e76651.
15. Mealing S, Barcena L, Hawkins N, Clark J, Eaton V, Hirji I, et al. The relative efficacy of imatinib, dasatinib and nilotinib for newly diagnosed chronic myeloid leukemia: a systematic review and network meta-analysis. Exp Hematol Oncol. 2013; 2: 5.
16. Wu MS, Tan SC, Xiong T. Indirect comparison of randomised controlled trials: comparative efficacy of dexlansoprazole vs. esomeprazole in the treatment of gastro-oesophageal reflux disease. Aliment Pharmacol Ther. 2013; 38: 190-201.
17. Dogliotti A, Paolasso E, Giugliano RP. Current and new oral antithrombotics in non-valvular atrial fibrillation: a network meta-analysis of 79 808 patients. Heart. 2014; 100: 396-405.
18. Kriston L, von Wolff A, Westphal A, Hölzel LP, Härter M. Efficacy and acceptability of acute treatments for persistent depressive disorder: a network meta-analysis. Depress Anxiety. 2014; 31: 621-30.
19. Roskell NS, Setyawan J, Zimovetz EA, Hodgkins P. Systematic evidence synthesis of treatments for ADHD in children and adolescents: indirect treatment comparisons of lisdexamfetamine with methylphenidate and atomoxetine. Curr Med Res Opin. 2014; 30: 1673-85.
20. Patel DA, Snedecor SJ, Tang WY, Sudharshan L, Lim JW, Cuffe R, et al. 48-week efficacy and safety of dolutegravir relative to commonly used third agents in treatment-naive HIV-1-infected patients: a systematic review and network meta-analysis. PLoS One. 2014; 9: e105653.
21. Fournier M, Germe M, Theobald K, Scholz GH, Lehmacher W. Indirect comparison of lixisenatide versus neutral protamine Hagedorn insulin as add-on to metformin and sulphonylurea in patients with type 2 diabetes mellitus. Ger Med Sci. 2014; 12: Doc14.
22. Palmer SC, Saglimbene V, Mavridis D, Salanti G, Craig JC, Tonelli M, et al. Erythropoiesis-stimulating agents for anaemia in adults with chronic kidney disease: a network meta-analysis. Cochrane Database Syst Rev. 2014; (12): CD010590.
23. Mantha S, Ansell J. Indirect comparison of dabigatran, rivaroxaban, apixaban and edoxaban for the treatment of acute venous thromboembolism. J Thromb Thrombolysis. 2015; 39: 155-65.
24. Singh S, Garg SK, Pardi DS, Wang Z, Murad MH, Loftus EV Jr. Comparative efficacy of pharmacologic interventions in preventing relapse of Crohn's disease after surgery: a systematic review and network meta-analysis. Gastroenterology. 2015; 148: 64-76.e2.
25. Linde K, Kriston L, Rücker G, Jamil S, Schumann I, Meissner K, et al. Efficacy and acceptability of pharmacological treatments for depressive disorders in primary care: systematic review and network meta-analysis. Ann Fam Med. 2015; 13: 69-79.
26. Bow EJ, Vanness DJ, Slavin M, Cordonnier C, Cornely OA, Marks D, et al. Systematic review and mixed treatment comparison meta-analysis of randomized clinical trials of primary oral antifungal prophylaxis in allogeneic hematopoietic cell transplant recipients. BMC Infect Dis. 2015; 15: 128.
27. Tramacere I, Del Giovane C, Salanti G, D'Amico R, Filippini G. Immunomodulators and immunosuppressants for relapsing-remitting multiple sclerosis: a network meta-analysis. Cochrane Database Syst Rev. 2015; (9): CD011381.
28. Miligkos M, Papamichael K, Vande Casteele N, Mantzaris GJ, Gils A, Levesque BG, et al. Efficacy and Safety Profile of Anti-tumor Necrosis Factor-α Versus Anti-integrin Agents for the Treatment of Crohn's Disease: A Network Meta-analysis of Indirect Comparisons. Clin Ther. 2016; 38: 1342-1358.e6.
29. Vieira MC, Kumar RN, Jansen JP. Comparative effectiveness of efavirenz, protease inhibitors, and raltegravir-based regimens as first-line treatment for HIV-infected adults: a mixed treatment comparison. HIV Clin Trials. 2011; 12: 175-89.

**Continuous outcome**

1. Wandel S, Jüni P, Tendal B, Nüesch E, Villiger PM, Welton NJ, Reichenbach S, Trelle S. Effects of glucosamine, chondroitin, or placebo in patients with osteoarthritis of hip or knee: network meta-analysis. BMJ. 2010; 341: c4675.
2. Stowe R, Ives N, Clarke CE, Handley K, Furmston A, Deane K, van Hilten JJ, Wheatley K, Gray R. Meta-analysis of the comparative efficacy and safety of adjuvant treatment to levodopa in later Parkinson's disease. Mov Disord. 2011; 26(4): 587-98.
3. Cipriani A, Barbui C, Salanti G, Rendell J, Brown R, Stockton S, Purgato M, Spineli LM, Goodwin GM, Geddes JR. Comparative efficacy and acceptability of antimanic drugs in acute mania: a multiple-treatments meta-analysis. Lancet. 2011; 378(9799): 1306-15.
4. Squires H, Simpson E, Meng Y, Harnan S, Stevens J, Wong R, Thomas S, Michaels J, Stansby G. A systematic review and economic evaluation of cilostazol, naftidrofuryl oxalate, pentoxifylline and inositol nicotinate for the treatment of intermittent claudication in people with peripheral arterial disease. Health Technol Assess. 2011; 15(40): 1-210.
5. Schwingshackl L, Missbach B, Dias S, König J, Hoffmann G. Impact of different training modalities on glycaemic control and blood lipids in patients with type 2 diabetes: a systematic review and network meta-analysis. Diabetologia. 2014; 57(9): 1789-97.
