# Supplementary material for: How robust are findings of pairwise and network meta-analysis in the presence of missing participant outcome data?
Source: BMC Med. 2021 Dec 21;19:323. doi: 10.1186/s12916-021-02195-y (PMC8691029; doi:10.1186/s12916-021-02195-y)
Supplement: Supplementary file 3 — Additional file 3. Note S1. Specification of the Bayesian models. Note S2. Exclusion due to convergence issues. [file 12916_2021_2195_MOESM3_ESM.docx]

**Additional file 3**

**Supplementary methods for the manuscript entitled “How robust are findings of pairwise and network meta-analysis in the presence of missing participant outcome data?”**

Loukia M. Spineli^1^, Chrysostomos Kalyvas^2^, Katerina Papadimitropoulou^3,4^

^1^Midwifery Research and Education Unit, Hannover Medical School, Hannover, Germany

^2^Biostatistics and Research Decision Sciences, MSD Europe Inc., Brussels, Belgium

^3^Clinical Epidemiology, Leiden University Medical Center, Leiden, The Netherlands

^4^Data Science and Biometrics, Danone Nutricia Research, Utrecht, The Netherlands

## Note S1. Specification of the Bayesian models

We used Bayesian methods to perform pairwise meta-analysis (PMA) and network meta-analysis (NMA) for offering flexibility in modelling observed and missing outcomes in the study-arms simultaneously in a single analysis. Furthermore, the Bayesian framework allows the incorporation of external information on the extent of between-study heterogeneity for a specific outcome and intervention-comparison type in the form of a prior distribution. This facilitates the estimation of the between-study variance ($\tau^{2}$) when the available evidence is scarce. We applied one-stage random-effects PMA/NMA. We incorporated the pattern-mixture model and the informative missingness odds ratio (IMOR) parameter to obtain the posterior mean of odds ratio (OR) in the logarithmic scale and its posterior standard deviation for the binary outcomes [11,13]. For the continuous outcomes, we applied one-stage random-effects PMA/NMA with pattern-mixture model and the informative missingness difference of means (IMDoM) parameter to obtain the posterior mean of standardised mean difference (SMD) and its posterior standard deviation [14]. The IMOR and IMDoM parameters are intuitively related to the OR and SMD, respectively [11,12]. In the NMA model, we incorporated the consistency equations and properly accommodated multi-arm studies [15].

We considered non-informative normal prior distribution with zero mean and variance equal to 10,000 for the location parameters (i.e., the underlying log odds and standardised mean in the control arm of each study and the underlying summary effect estimate) [15]. We assigned empirically-based prior distributions on $\tau^{2}$ specific to the investigated outcome and intervention-comparison type following Turner et al. [16] for binary outcomes and Rhodes et al. [17] for continuous outcomes. We used JAGS (version 4.3.0) via the R-package R2jags (statistical software R, version 4.0.4) to perform Bayesian PMA and NMA [18–20]. We ran three chains of different initial values with 100,000 iterations and 10,000 burn-in. Due to the number of models and their parameters, we used a pragmatic approach to assess their convergence. We inferred convergence when the Gelman–Rubin convergence diagnostic of a given parameter did not exceed 1.1 [21].

**Note S2. Exclusion due to convergence issues**

We excluded one PMA on a binary outcome from the subsequent analyses, as the summary log OR failed to converge [33]. Convergence was not achieved even after applying a weakly informative prior on the summary log OR following the suggestion of Günhan et al. [35] for a random-effects meta-analysis of a few studies with rare events. This PMA had zero non-events in the control arm of all three studies (Additional file 5: Table S1). We also excluded one NMA on a binary outcome due to convergence issues in the summary log OR of all comparisons with no treatment [34]. This intervention was included only in one study and had zero non-events (Additional file 5: Table S2). Therefore, the final analyses were based on 107 PMAs (94 on binary and 13 on continuous primary outcomes) and 33 NMAs (28 on binary and five on continuous primary outcomes).
