# Supplementary material for: How robust are findings of pairwise and network meta-analysis in the presence of missing participant outcome data?
Source: BMC Med. 2021 Dec 21;19:323. doi: 10.1186/s12916-021-02195-y (PMC8691029; doi:10.1186/s12916-021-02195-y)
Supplement: Supplementary file 4 — Additional file 4. Supplementary figures. [file 12916_2021_2195_MOESM4_ESM.docx]

**Additional file 4**

**Supplementary figures for the manuscript entitled “How robust are findings of pairwise and network meta-analysis in the presence of missing participant outcome data?”**

Loukia M. Spineli^1^, Chrysostomos Kalyvas^2^, Katerina Papadimitropoulou^3,4^

^1^Midwifery Research and Education Unit, Hannover Medical School, Hannover, Germany

^2^Biostatistics and Research Decision Sciences, MSD Europe Inc., Brussels, Belgium

^3^Clinical Epidemiology, Leiden University Medical Center, Leiden, The Netherlands

^4^Data Science and Biometrics, Danone Nutricia Research, Utrecht, The Netherlands

**
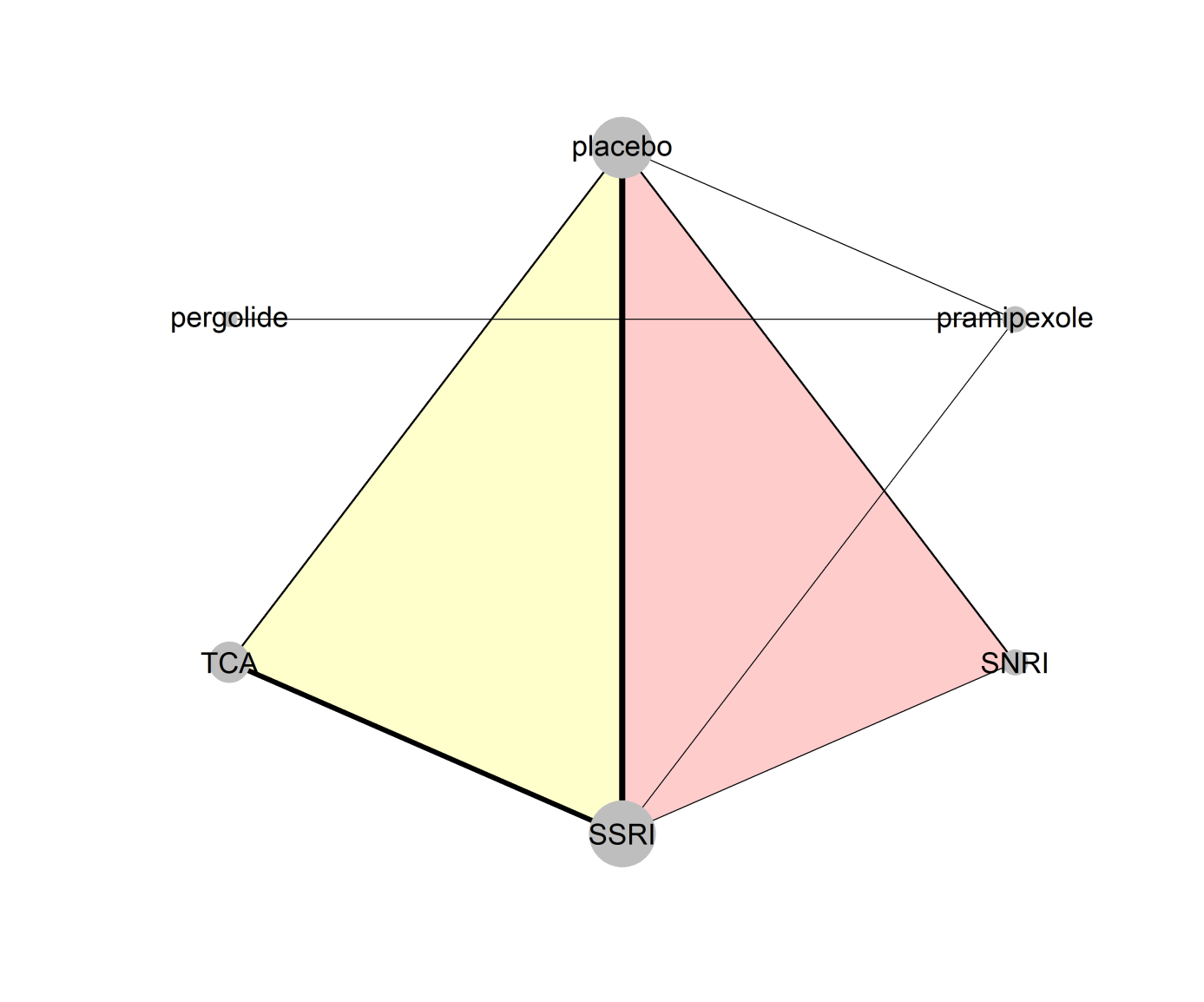
**

**Figure S1.** A network of five antidepressants and a placebo for the symptom relief of depression in patients with Parkinson's disease [23]. The size of the nodes is proportional to the number of observed treatment comparisons. The thickness of the edge is proportional to the number of studies that investigated that comparison. The coloured triangles indicate comparisons informed by multi-arm studies.


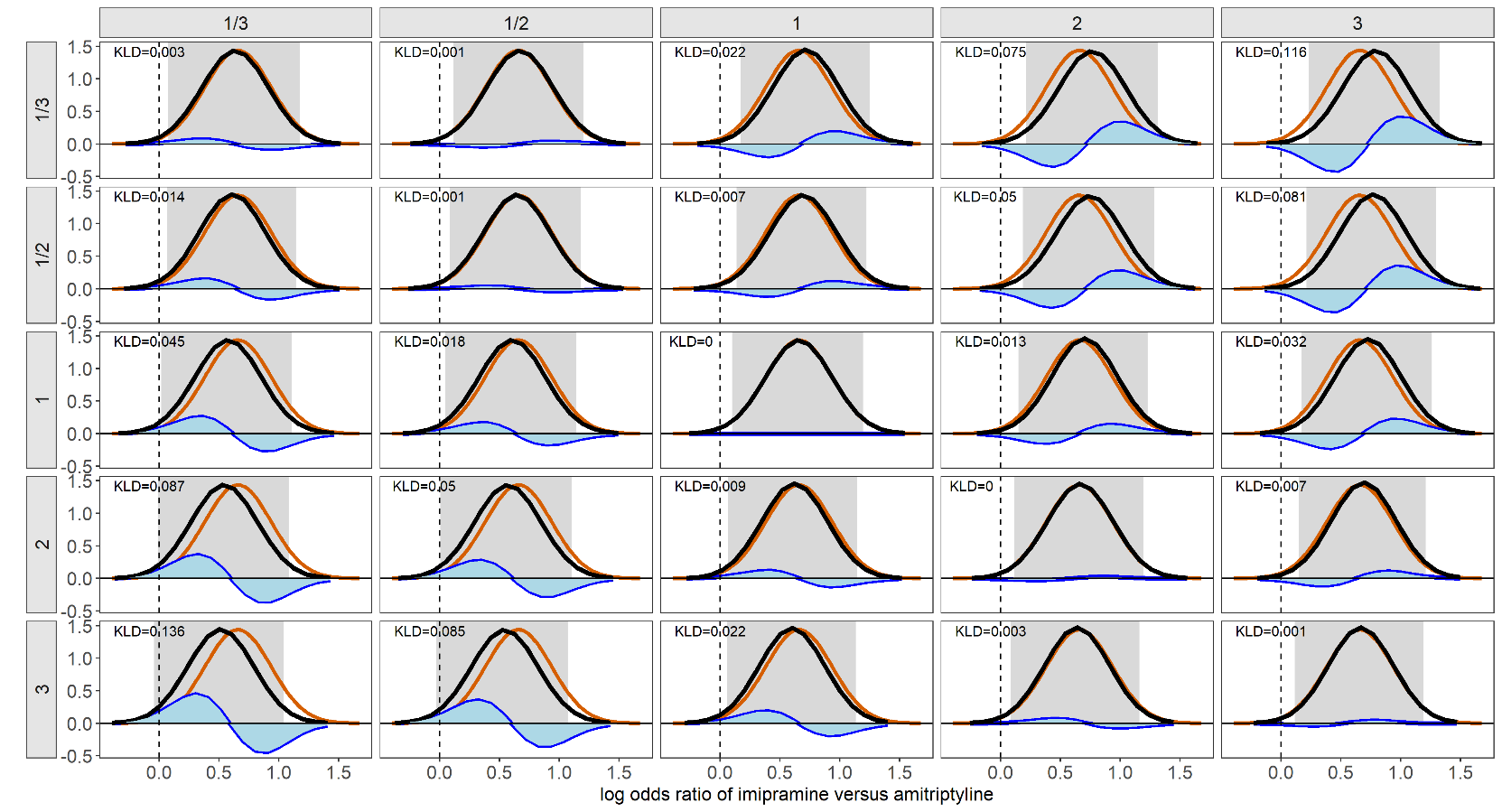
**Figure S2.** A panel of density plots on the summary log odds ratio (OR) of imipramine versus amitriptyline from Guaiana et al. [37]. The red line indicates the posterior distribution of the log OR under the primary analysis. The black lines indicate the posterior distribution of the log OR under the alternative re-analyses. The alternative re-analyses refer to 24 different assumptions about the informative missingness difference of means parameter in imipramine (facets at the top of the panel) and amitriptyline (facets at the left of the panel). The blue area corresponds to the Kullback-Leibler divergence (KLD) measure. The vertical dotted line refers to log OR equal to zero (no difference). The grey rectangular indicates the 95% credible interval of log OR under the corresponding re-analysis.


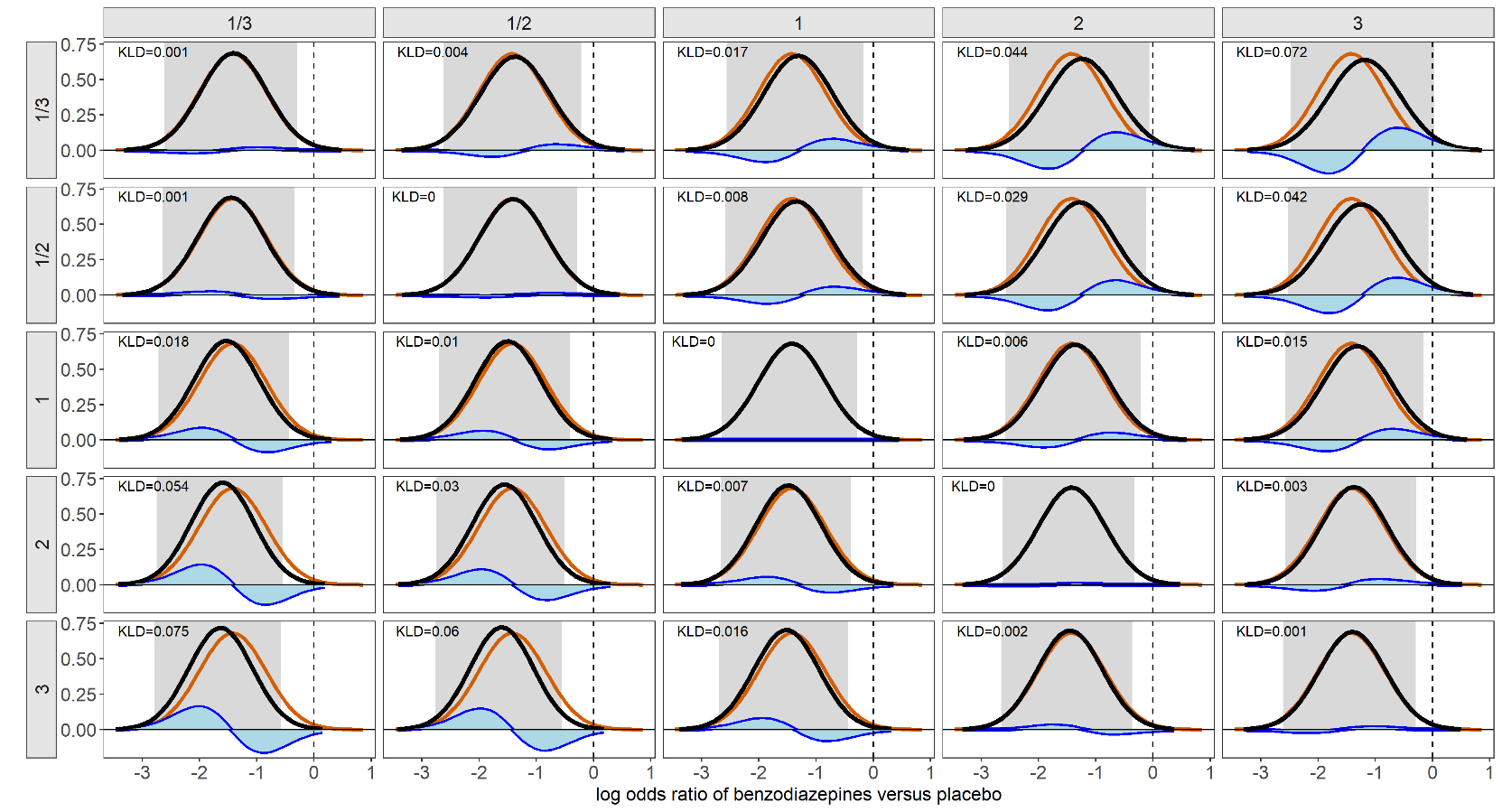
**Figure S3.** A panel of density plots on the summary log odds ratio (OR) of benzodiazepines versus placebo from Dold et al. [38]. The red line indicates the posterior distribution of the log OR under the primary analysis. The black lines indicate the posterior distribution of the log OR under the alternative re-analyses. The alternative re-analyses refer to 24 different assumptions about the informative missingness difference of means parameter in benzodiazepines (facets at the top of the panel) and placebo (facets at the left of the panel). The blue area corresponds to the Kullback-Leibler divergence (KLD) measure. The vertical dotted line refers to log OR equal to zero (no difference). The grey rectangular indicates the 95% credible interval of log OR under the corresponding re-analysis.


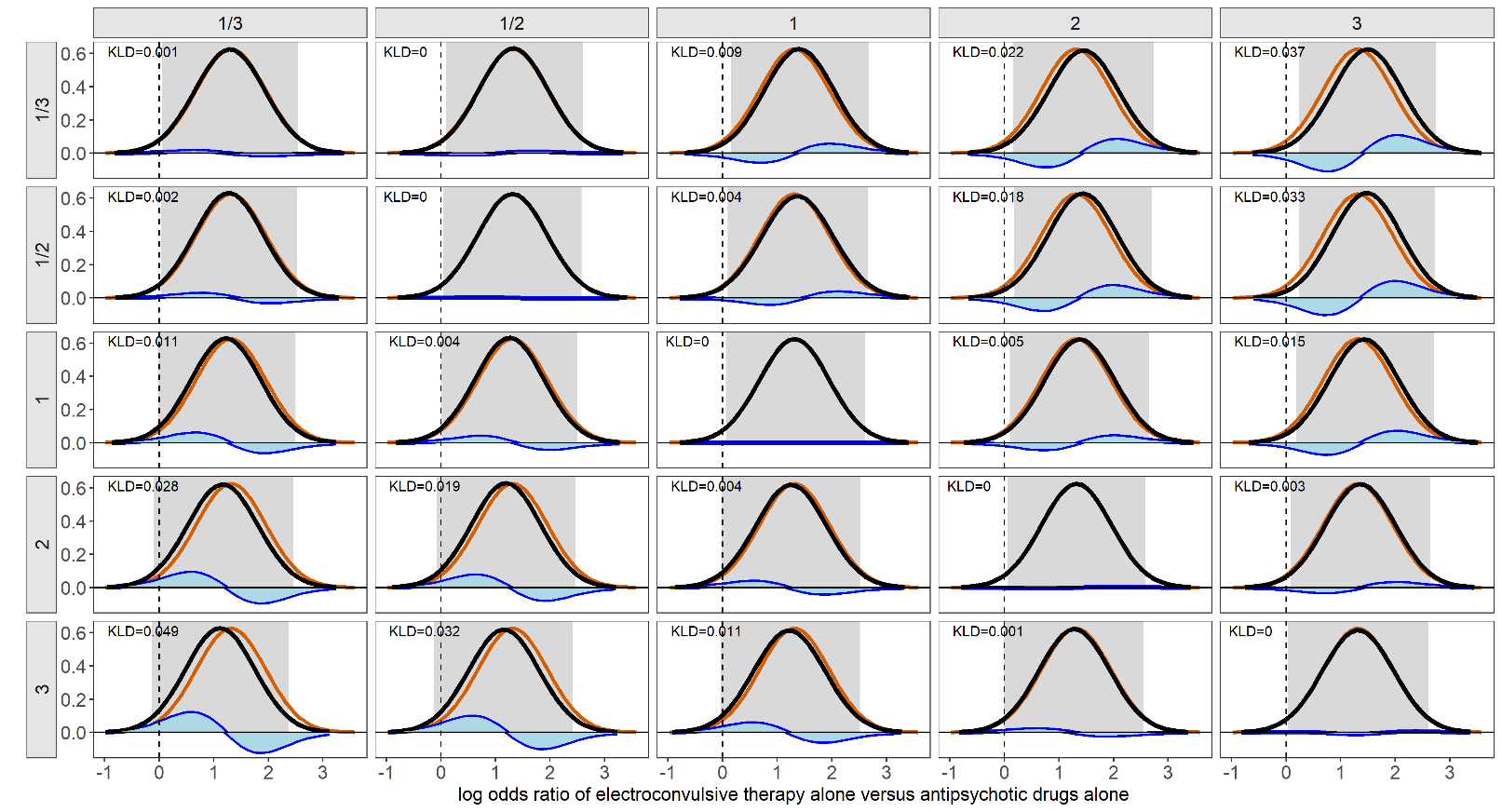
**Figure S4.** A panel of density plots on the summary log odds ratio (OR) of electroconvulsive therapy alone versus antipsychotic drugs alone from Tharyan et al. [39]. The red line indicates the posterior distribution of the log OR under the primary analysis. The black lines indicate the posterior distribution of the log OR under the alternative re-analyses. The alternative re-analyses refer to 24 different assumptions about the informative missingness difference of means parameter in electroconvulsive therapy alone (facets at the top of the panel) and antipsychotic drugs alone (facets at the left of the panel). The blue area corresponds to the Kullback-Leibler divergence (KLD) measure. The vertical dotted line refers to log OR equal to zero (no difference). The grey rectangular indicates the 95% credible interval of log OR under the corresponding re-analysis.


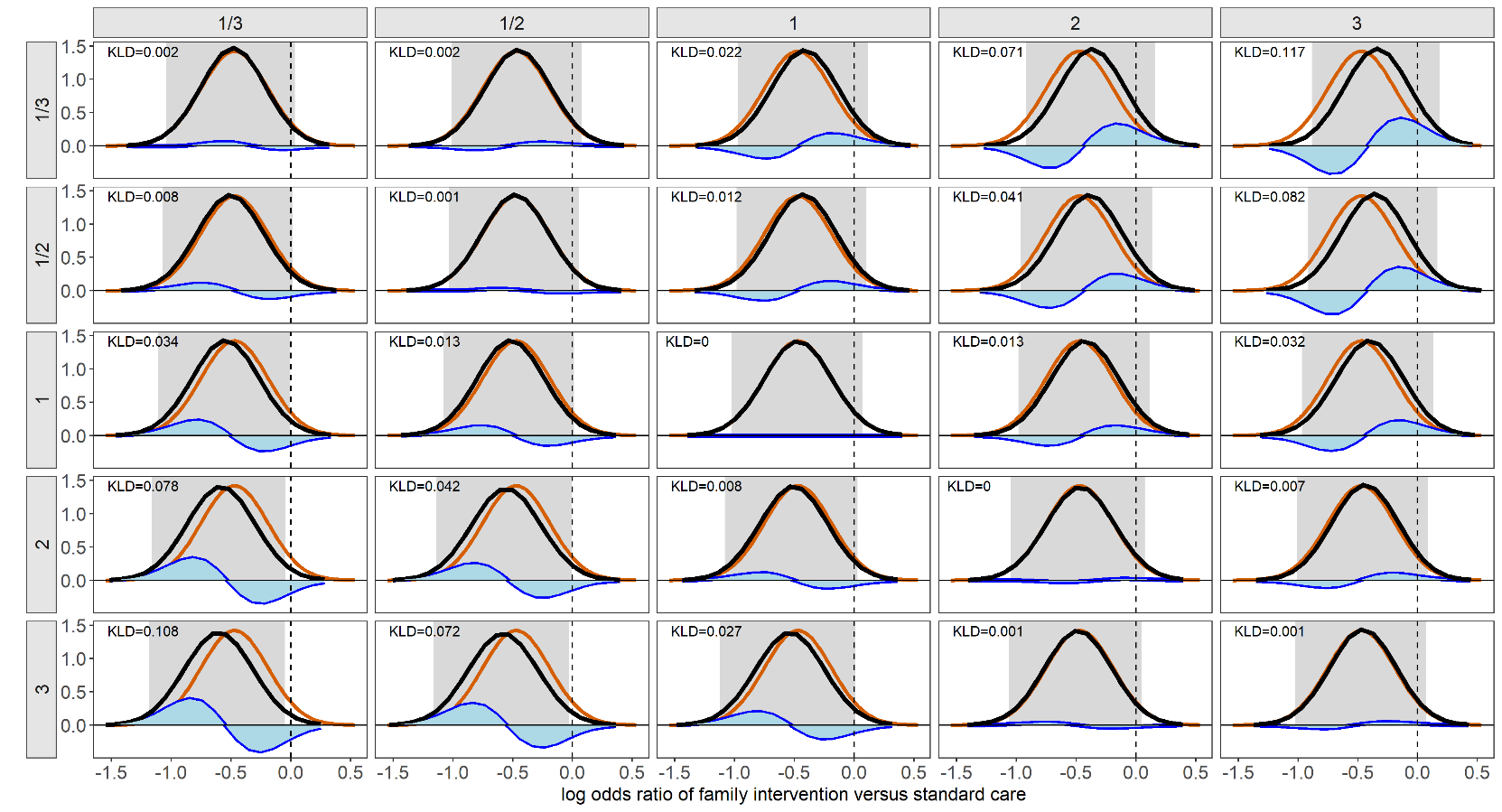
**Figure S5.** A panel of density plots on the summary log odds ratio (OR) of family intervention versus standard care from Pharoah et al. [40]. The red line indicates the posterior distribution of the log OR under the primary analysis. The black lines indicate the posterior distribution of the log OR under the alternative re-analyses. The alternative re-analyses refer to 24 different assumptions about the informative missingness difference of means parameter in family intervention (facets at the top of the panel) and standard care (facets at the left of the panel). The blue area corresponds to the Kullback-Leibler divergence (KLD) measure. The vertical dotted line refers to log OR equal to zero (no difference). The grey rectangular indicates the 95% credible interval of log OR under the corresponding re-analysis.


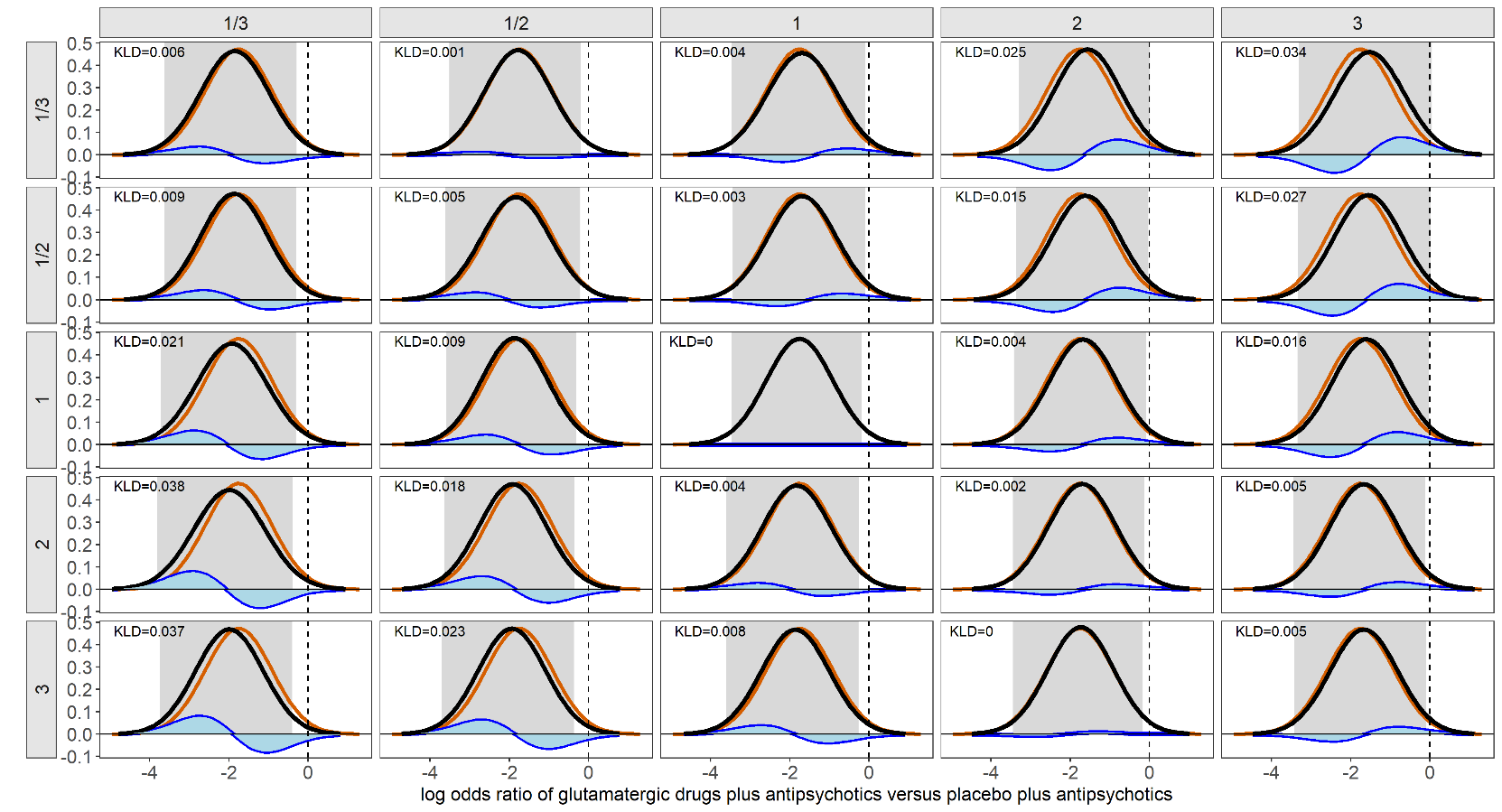
**Figure S6.** A panel of density plots on the summary log odds ratio (OR) of glutamatergic drugs plus antipsychotics versus placebo plus antipsychotics from Tuominen et al. [41]. The red line indicates the posterior distribution of the log OR under the primary analysis. The black lines indicate the posterior distribution of the log OR under the alternative re-analyses. The alternative re-analyses refer to 24 different assumptions about the informative missingness difference of means parameter in glutamatergic drugs plus antipsychotics (facets at the top of the panel) and placebo plus antipsychotics (facets at the left of the panel). The blue area corresponds to the Kullback-Leibler divergence (KLD) measure. The vertical dotted line refers to log OR equal to zero (no difference). The grey rectangular indicates the 95% credible interval of log OR under the corresponding re-analysis.


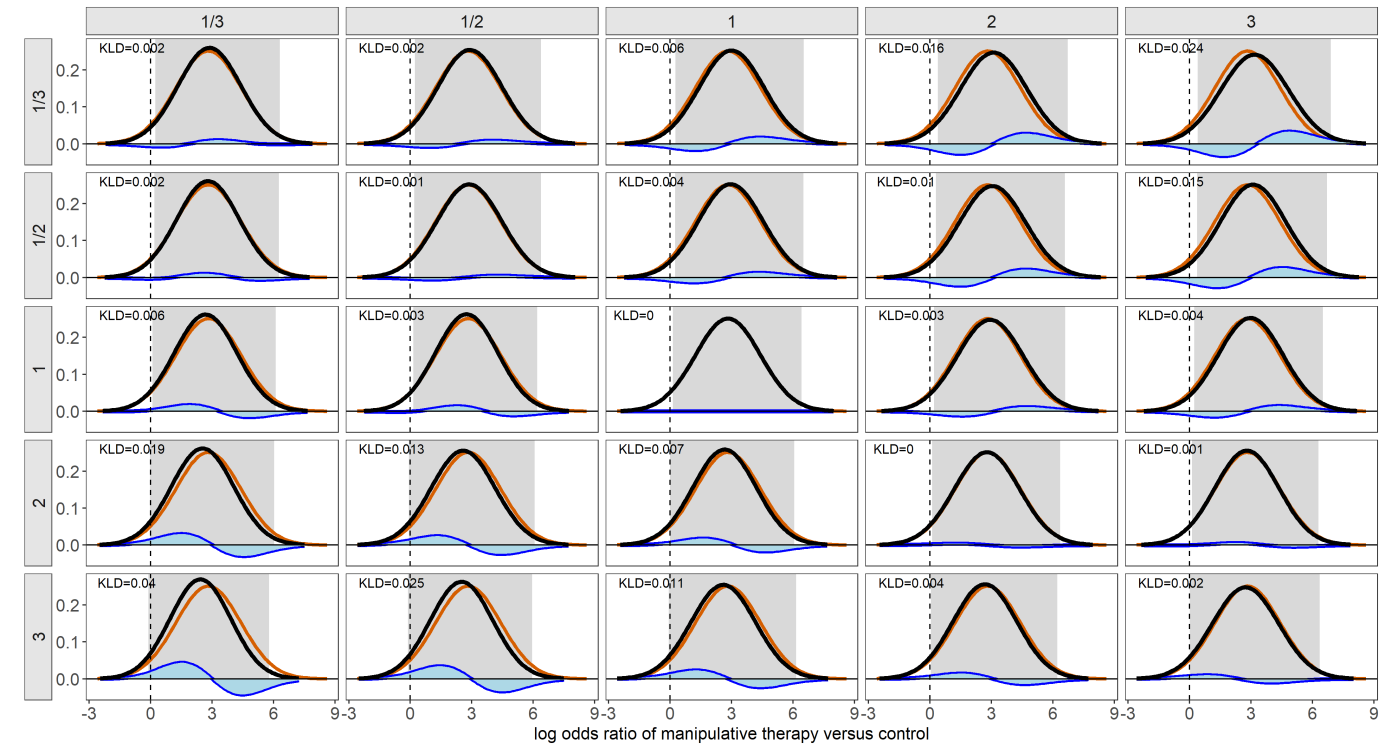
**Figure S7.** A panel of density plots on the summary log odds ratio (OR) of manipulative therapy versus control from Dobson et al. [42]. The red line indicates the posterior distribution of the log OR under the primary analysis. The black lines indicate the posterior distribution of the log OR under the alternative re-analyses. The alternative re-analyses refer to 24 different assumptions about the informative missingness difference of means parameter in manipulative therapy (facets at the top of the panel) and control (facets at the left of the panel). The blue area corresponds to the Kullback-Leibler divergence (KLD) measure. The vertical dotted line refers to log OR equal to zero (no difference). The grey rectangular indicates the 95% credible interval of log OR under the corresponding re-analysis.


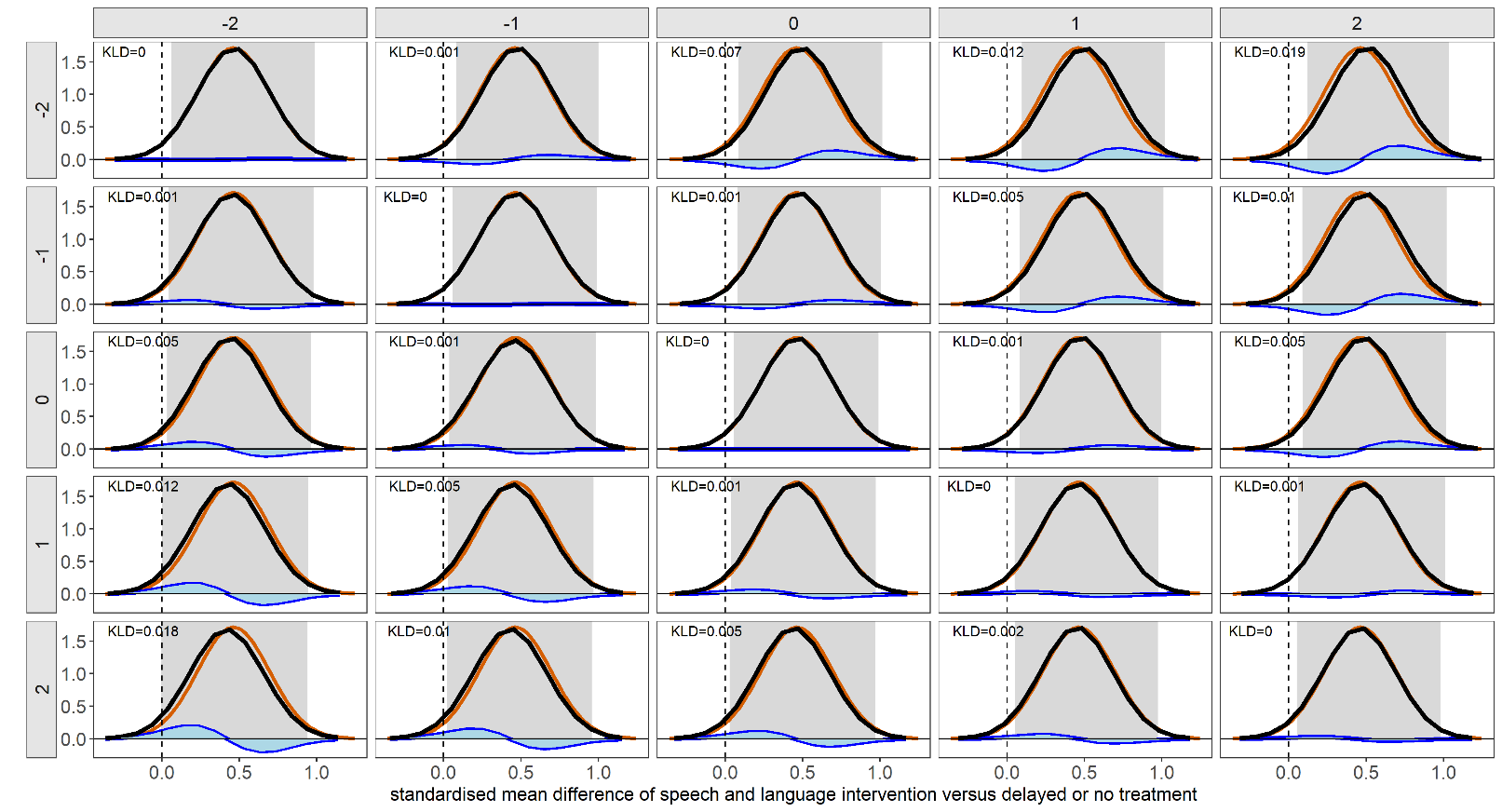
**Figure S8.** A panel of density plots on the summary standardised mean difference (SMD) of speech and language intervention versus delayed or no treatment from Law et al. [43]. The red line indicates the posterior distribution of the SMD under the primary analysis. The black lines indicate the posterior distribution of the SMD under the alternative re-analyses. The alternative re-analyses refer to 24 different assumptions about the informative missingness difference of means parameter in speech and language intervention (facets at the top of the panel) and delayed or no treatment (facets at the left of the panel). The blue area corresponds to the Kullback-Leibler divergence (KLD) measure. The vertical dotted line refers to SMD equal to zero (no difference). The grey rectangular indicates the 95% credible interval of SMD under the corresponding re-analysis.


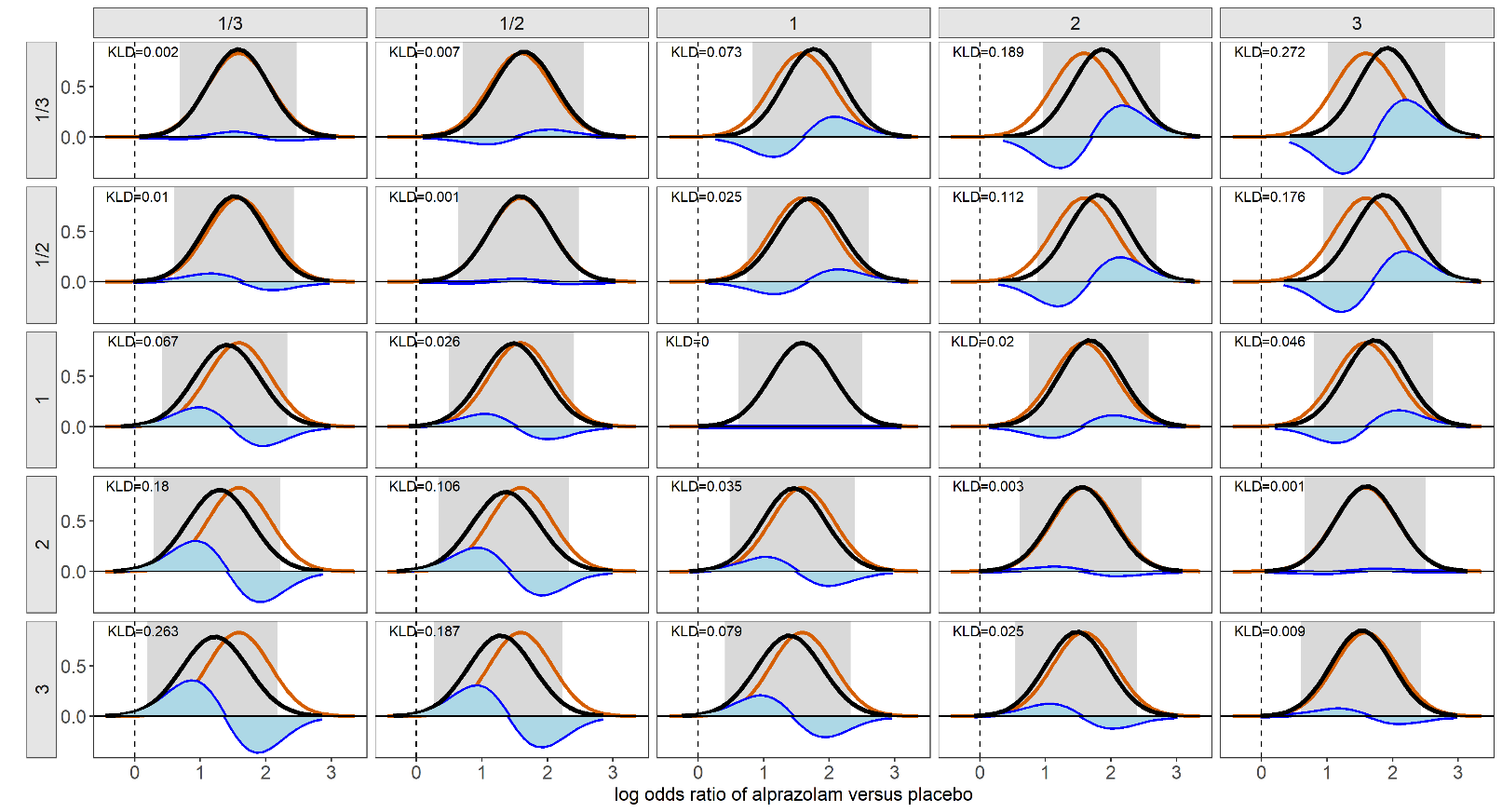
**Figure S9.** A panel of density plots on the summary log odds ratio (OR) of alprazolam versus placebo from van Marwijk et al. [44]. The red line indicates the posterior distribution of the log OR under the primary analysis. The black lines indicate the posterior distribution of the log OR under the alternative re-analyses. The alternative re-analyses refer to 24 different assumptions about the informative missingness difference of means parameter in alprazolam (facets at the top of the panel) and placebo (facets at the left of the panel). The blue area corresponds to the Kullback-Leibler divergence (KLD) measure. The vertical dotted line refers to log OR equal to zero (no difference). The grey rectangular indicates the 95% credible interval of log OR under the corresponding re-analysis.
