# Supplementary material for: How robust are findings of pairwise and network meta-analysis in the presence of missing participant outcome data?
Source: BMC Med. 2021 Dec 21;19:323. doi: 10.1186/s12916-021-02195-y (PMC8691029; doi:10.1186/s12916-021-02195-y)
Supplement: Supplementary file 5 — Additional file 5. Supplementary tables. [file 12916_2021_2195_MOESM5_ESM.docx]

**Additional file 5**

**Supplementary tables for the manuscript entitled “How robust are findings of pairwise and network meta-analysis in the presence of missing participant outcome data?”**

Loukia M. Spineli^1^, Chrysostomos Kalyvas^2^, Katerina Papadimitropoulou^3,4^

^1^Midwifery Research and Education Unit, Hannover Medical School, Hannover, Germany

^2^Biostatistics and Research Decision Sciences, MSD Europe Inc., Brussels, Belgium

^3^Clinical Epidemiology, Leiden University Medical Center, Leiden, The Netherlands

^4^Data Science and Biometrics, Danone Nutricia Research, Utrecht, The Netherlands

| **Table S1. Subgroup 1 of Analysis 1.1 in the Cochrane systematic review of Marques et al. [33]†** | | | | | | | | |
| --- | --- | --- | --- | --- | --- | --- | --- | --- |
| **Study** | **Trifluoperazine** | | | | **Placebo** | | | |
|  | $\boldsymbol{r}_{\boldsymbol{E}}$ | $\boldsymbol{f}_{\boldsymbol{E}}$ | $\boldsymbol{m}_{\boldsymbol{E}}$ | $\boldsymbol{n}_{\boldsymbol{E}}$ | $\boldsymbol{r}_{\boldsymbol{C}}$ | $\boldsymbol{f}_{\boldsymbol{C}}$ | $\boldsymbol{m}_{\boldsymbol{C}}$ | $\boldsymbol{n}_{\boldsymbol{C}}$ |
| Bishop, 1964 | 9 | 5 | 0 | 14 | 14 | 0 | 0 | 14 |
| Clark, 1975 | 8 | 3 | 3 | 14 | 12 | 0 | 1 | 13 |
| Menon, 1972 | 12 | 8 | 0 | 20 | 20 | 0 | 0 | 20 |
| $r_{k}$, number of observed events; $f_{k}$, number of observed non-events; $m_{k}$, number of missing participants; $n_{k}$, number of randomised participants in arm $k$ with $k=E$(xperimental), C(ontrol).  †Missing participants were extracted from subgroup 1 (any reason) of Analysis 1.9. For the studies Bishop and Menon, we referred to the ‘Characteristics of the studies’ to gain insight into the number of missing participants: the intention-to-treat approach was considered without information on the assumptions used to impute the missing participants in either arm. | | | | | | | | |

| **Table S2. Efficacy of several ESAs to prevent blood transfusion (Analysis 1.1) by Palmer et al. [34]†** | | | | | | | | |
| --- | --- | --- | --- | --- | --- | --- | --- | --- |
| **Study** | **Comparison** | | **Baseline arm** | | | **Non-baseline arm** | | |
|  | $\boldsymbol{t}_{\boldsymbol{1}}$ | $\boldsymbol{t}_{\boldsymbol{2}}$ | $\boldsymbol{r}_{\boldsymbol{1}}$ | $\boldsymbol{m}_{\boldsymbol{1}}$ | $\boldsymbol{n}_{\boldsymbol{1}}$ | $\boldsymbol{r}_{\boldsymbol{2}}$ | $\boldsymbol{m}_{\boldsymbol{2}}$ | $\boldsymbol{n}_{\boldsymbol{2}}$ |
| Canadian EPO Study 1990 | A | F | 9 | 8 | 40 | 65 | 11 | 78 |
| Roth 1994 | A | F | 6 | 25 | 40 | 16 | 23 | 43 |
| Bennett 1991 | A | G | 39 | 1 | 41 | 82 | 8 | 90 |
| Bahlmann 1991 | A | G | 18 | 20 | 66 | 48 | 10 | 63 |
| TREAT Study 2005 | A | C | 1366 | 164 | 2026 | 1652 | 153 | 2102 |
| Patel 2012 | B | F | 29 | 10 | 39 | 87 | 27 | 118 |
| Akizawa 2011 | C | F | 117 | 43 | 161 | 111 | 49 | 160 |
| Nissenson 2002 | C | F | 294 | 27 | 338 | 247 | 54 | 338 |
| Krivoshiev 2010 | D | F | 151 | 78 | 232 | 164 | 65 | 230 |
| Krivoshiev 2008 | D | F | 263 | 32 | 305 | 249 | 46 | 304 |
| Martin 2007 | D | F | 384 | 130 | 560 | 142 | 39 | 192 |
| AMICUS Study 2007 | E | G | 125 | 3 | 135 | 41 | 3 | 46 |
| TIVOLI Study 2013 | E | C | 44 | 1 | 46 | 22 | 3 | 25 |
| ARCTOS Study 2008 | E | C | 141 | 17 | 162 | 140 | 11 | 162 |
| CORDATUS Study 2011 | E | C | 132 | 12 | 154 | 132 | 17 | 154 |
| PATRONUS Study 2010 | E | C | 148 | 58 | 245 | 116 | 97 | 245 |
| A, placebo; B, no treatment; C, darbepoetin-alfa; D, biosimilar-ESA; E, methoxy-polyethylene-glycol-epoetin-beta; ESA, erythropoiesis-stimulating agents; F, epoetin-alfa; G, epoetin-beta; $r_{k}$, number of observed events; $m_{k}$, number of missing participants; $n_{k}$, number of randomised participants in arm $k$ with $k=1,2$.  †The outcome has been recorded so that OR more than 1 indicates beneficial effect for the first intervention in each comparison ($t_{2}$ versus $t_{1}$). We referred to the ‘Characteristics of the studies’ to extract the number of missing participants, where possible. Missing participants could not be extracted for three studies, and we excluded these studies from our analysed dataset. | | | | | | | | |

| **Table S3. Characteristics of the PMA and NMAs with counterintuitive results^†^** | | | | |
| --- | --- | --- | --- | --- |
| ID* | Number of studies | % total response | % total MOD | total sample |
| PMAs with binary outcome in the 'low risk' group | | | | |
| 5^th^ | 25 | 42 (5, 63) | 0 (0, 36) | 55 (20, 229) |
| 7^th^ | 10 | 20 (8, 45) | 7 (0, 40) | 52 (20, 190) |
| 24^th^ | 23 | 45 (10, 74) | 10 (0, 49) | 42 (12, 177) |
| 84^th^ | 9 | 50 (29, 74) | 2 (0, 25) | 107 (35, 1362) |
| PMAs with continuous outcome in the 'low risk' group | | | | |
| 2^nd^ | 8 | NA | 0 (0, 25) | 25 (5, 59) |
| 10^th^ | 3 | NA | 0 (0, 31) | 42 (12, 60) |
| NMAs with binary outcome in the 'low risk' group | | | | |
| 1^st^ | 104 | 61 (11, 89) | 4 (0, 24) | 237 (22, 708) |
| 28^th^ | 13 | 48 (30, 75) | 4 (0, 19) | 339 (90, 905) |
| 29^th^ | 7 | 80 (72, 87) | 0 (0, 15) | 563 (79, 883) |
| (all) NMAs with continuous outcome in the 'low risk' group | | | | |
| 1^st^ | 7 | NA | 0 (0, 4) | 222 (202, 622) |
| 2^nd^ | 29 | NA | 6 (0, 56) | 197 (37, 472) |
| 3^rd^ | 62 | NA | 2 (0, 50) | 240 (17, 680) |
| 4^th^ | 8 | NA | 0 (0, 19) | 179 (106, 785) |
| 5^th^ | 14 | NA | 1 (0, 31) | 41 (19, 221) |
| PMAs with binary outcome in the 'high risk' group | | | | |
| 27^th^ | 4 | 55 (20, 62) | 29 (13, 41) | 56 (27, 76) |
| 87^th^ | 3 | 16 (14, 72) | 21 (0, 30) | 29 (20, 86) |
| PMAs with continuous outcome in the 'high risk' group | | | | |
| 5^th^ | 3 | NA | 40 (39, 41) | 82 (82, 102) |
|  | | | | |
| NA, not applicable; NMA, network meta-analysis; MOD, missing participant outcome data; PMA, pairwise meta-analysis.  ^†^Results are median (minimum, maximum)  *Identification numbers refer to the analyses in the Additional files 1 and 2. | | | | |
